# Quality of Life in Autism: A Systematic Review and Meta‐Analysis of Patients and Caregivers

**DOI:** 10.1002/aur.70348

**Published:** 2026-08-24

**Authors:** Kamila Castro, Camila Kümmel Duarte, Eduarda Silva, Laura Hoffmann, Jenifer Lopes Borchardt, Laura Pohl, Josiane Luçardo, Myrella Lapschies, Andreia Morales Cascaes, Ana Amélia Freitas‐Vilela, Adriane Cenci, Juliana S. Vaz

**Affiliations:** ^1^ Graduate Program in Nutrition and Food Federal University of Pelotas (UFPel) Pelotas Rio Grande do Sul Brazil; ^2^ Department of Nutrition, School of Nursing Federal University of Minas Gerais (UFMG) Belo Horizonte Minas Gerais Brazil; ^3^ Center for the Arts Federal University of Pelotas (UFPel) Pelotas Rio Grande do Sul Brazil; ^4^ Department of Public Health Federal University of Santa Catarina (UFSC) Florianópolis Santa Catarina Brazil; ^5^ Faculty of Medicine Federal University of Jataí (UFJ) Jataí Goiás Brazil; ^6^ Education Center Federal University of Rio Grande do Norte (UFRN) Natal Rio Grande do Norte Brazil; ^7^ Faculty of Nutrition Federal University of Pelotas (UFPel) Pelotas Rio Grande do Sul Brazil

## Abstract

Autism spectrum disorder (ASD) is a lifelong neurodevelopmental condition associated with significant challenges in daily functioning and well‐being. Caregivers often experience substantial psychological and physical burden. However, comprehensive synthesis of quality of life (QoL) outcomes for both individuals with ASD and their caregivers remains limited. This study evaluated QoL in individuals with ASD and their caregivers and examined key psychological, social, and economic determinants. A systematic review and meta‐analysis were conducted following PRISMA guidelines (PROSPERO: CRD42024538872). PubMed, Scopus, Embase, PsycINFO, and preprint repositories were searched for studies published between January 2013 and July 2025. Eligible studies assessed QoL using validated instruments in individuals with ASD aged ≤ 19 years and/or their caregivers. Random‐effects meta‐analysis was used to estimate pooled mean differences. Of 14,368 records identified, 216 studies were included. Quantitative meta‐analyses included up to 173 studies, depending on the outcome and availability of comparable data. Individuals with ASD scored 21.7 points lower than peers (95% CI: −26.9 to −16.5; *p* < 0.0001). Caregivers scored 13.64 points lower than controls (95% CI: −19.21 to −8.06; *p* < 0.0001). Emotional and social domains were most affected. Higher Human Development Index was associated with better QoL. QoL is markedly reduced in individuals with ASD and their caregivers, underscoring the need for multidimensional and equitable interventions.

## 1 Introduction

Autism spectrum disorder (ASD) is a neurodevelopmental condition characterized by persistent challenges in social communication, restricted interests, and repetitive behaviors (American Psychiatric Association 2013). Its clinical presentation is highly heterogeneous, encompassing a wide range of functional abilities, comorbidities, and support needs. Recent epidemiological data indicate that the global prevalence of ASD has increased substantially over the past decades, with current estimates suggesting that about one in 100 individuals worldwide are affected (World Health Organization (WHO) 2025). In the United States, the Centers for Disease Control and Prevention (CDC) report a prevalence of 1 in 31 children, reflecting improved diagnostic criteria and greater awareness (Shaw et al. 2025). Although ASD occurs across all ethnic and socioeconomic groups, significant disparities remain in timely diagnosis, access to interventions, and long‐term support (Shaw et al. 2025).

Quality of life (QoL) has emerged as a key outcome measure in autism research, encompassing physical, psychological, social, and environmental domains that contribute to overall well‐being. Individuals with ASD frequently experience challenges in emotional health, social participation, education, and autonomy, which can persist into adulthood when structured support systems often decline (Musetti et al. 2021). Assessing QoL in ASD presents important conceptual and methodological challenges. QoL is a multidimensional construct that may be perceived differently by individuals with ASD and by proxy respondents, such as parents or caregivers. While self‐reported measures provide direct insight into subjective well‐being, they may be influenced by communication abilities, cognitive characteristics, and individual perceptions. Conversely, proxy reports may capture observable functioning but can be affected by caregiver stress and expectations (Van Heijst and Geurts 2015).

Multiple studies have emphasized that beyond symptom management, improving QoL requires person‐centered approaches that enhance adaptive functioning, community inclusion, and self‐determination (Van Heijst and Geurts 2015). These difficulties can compromise their overall life satisfaction, particularly in adulthood, when structured support often diminishes (Ayres et al. 2018; Kuhlthau et al. 2010).

The impact of ASD extends beyond the affected individual, profoundly influencing the QoL of caregivers, most commonly parents (Musetti et al. 2021). Caregiving for a child with ASD is associated with increased physical and emotional strain, social isolation, and financial burden. Studies consistently indicate that caregivers of children with ASD report higher levels of stress, anxiety, and depressive symptoms compared to those caring for neurotypical children (Hayes and Watson 2013). Factors such as the severity of ASD symptoms, behavioral challenges, and access to health and social care services are critical determinants of caregivers' well‐being. These multidimensional stressors underscore the need for comprehensive assessments that capture not only child‐related outcomes but also caregiver health and functioning.

Several standardized instruments have been employed to assess patients' QoL, such as the Pediatric Quality of Life Inventory (PedsQL) (Varni et al. 2001), the child health questionnaire (CHQ) (Landgraf et al. 1996), and the KINDL‐R (Ravens‐Sieberer and Bullinger 1998). In addition, caregivers' QoL has been evaluated using instruments including the WHOQOL‐BREF (The Whoqol Group 1998) and the SF‐36 (Ware and Sherbourne 1992). These assessment tools facilitate a nuanced understanding of the specific challenges faced by patients and caregivers and inform the development of effective interventions.

Despite the growing body of literature on QoL in ASD, existing reviews have largely focused on specific subgroups (e.g., children or caregivers), single domains of QoL, or narrative syntheses without quantitative integration. Moreover, prior studies have rarely examined QoL in individuals with ASD and their caregivers within a unified analytical framework, limiting the understanding of how these experiences intersect. In addition, there is a lack of global comparative analyses exploring how contextual factors—such as socioeconomic development and geographic variation—shape QoL outcomes. These gaps restrict the ability to derive comprehensive and policy‐relevant conclusions from the existing evidence.

To address these limitations, the present study provides a comprehensive and quantitative synthesis of QoL in both individuals with ASD and their caregivers, integrating evidence across multiple domains and populations. By applying meta‐analytic techniques and incorporating contextual moderators such as Human Development Index (HDI) and geographic region, this review advances prior work by offering a global, multidimensional, and comparative perspective on QoL in ASD.

The objective of this systematic review is to evaluate the QoL of individuals diagnosed with ASD and their caregivers. Specifically, this review aims to examine the psychological, social, and economic determinants influencing QoL and to synthesize evidence from existing studies to identify major challenges and potential intervention targets. By providing a comprehensive analysis of the factors shaping well‐being in individuals with ASD and their caregivers, the study seeks to inform clinical practice, guide public health policies, and support the development of tailored interventions that promote better outcomes for both groups.

## 2 Methods

### 2.1 Protocol and Registration

This review was reported in accordance with the Preferred Reporting Items for Systematic Review and Meta‐Analyses (PRISMA) and followed the methodological guidance of the *Cochrane Handbook for Systematic Reviews of Interventions*. The protocol was registered in the International Prospective Register of Systematic Reviews (PROSPERO: CRD42024538872).

### 2.2 Search Strategy

The research question was “What is the quality of life experienced by children and adolescents diagnosed with autism spectrum disorder and their caregivers?” Later, a search strategy was created based on adapting the research question to the PECO strategy (Population, Exposure, Comparison, Outcome) (Appendix S1, Table S1). Because the primary objective of this review was to synthesize QoL outcomes among individuals with ASD and their caregivers, the presence of a comparison group was not required for study inclusion. The literature search was carried out in April 2024 and updated in June 2025. Databases (PubMed, SciELO, Scopus, Embase, and PsycINFO) and preprint repositories (BioRXiv, medRXiv, OSF, and Preprints.org) were consulted, using search keys composed of a combination of terms and indexed terms connected through “OR” and “AND” operators (Appendix S1, Table S2).

### 2.3 Eligibility Criteria

Studies assessing the QoL of children and adolescents with ASD and/or their caregivers/family members using validated instruments were included. Eligible study designs included clinical, cohort and instrument validation studies. Review studies, experimental studies with animal models, case reports, dissertations, theses, short communications, institutional reports, editorials, letters to the editor, comments and information from websites or blogs were excluded. In addition, studies including individuals with ASD over the age of 19 and studies in which teachers or therapists were considered caregivers were excluded. Studies including participants with ASD and other primary neurodevelopmental conditions (e.g., Down syndrome or cerebral palsy) were eligible only when results for the ASD subgroup were reported separately; otherwise, they were excluded.

### 2.4 Selection and Data Collection Process

Studies were imported into Rayyan web application to conduct the selection stages. After removing duplicates, the following screening stages were conducted by pairs of blinded reviewers: (i) title and abstract screening and (ii) full‐text screening. Inconsistencies in the selection between the pairs were resolved in meetings between the group of reviewers and the senior reviewer at the end of each stage. Only studies published in English, Portuguese, Spanish, or French were included due to translation feasibility. Trial registries were not searched.

A standardized spreadsheet designed for this review was used to extract data related to the characteristics of the selected studies (authorship, year, country, and participants), QoL assessment (instrument, domains assessed, and main results) and statistical analyses applied. The data were extracted by the independent reviewers and checked by the senior reviewer.

### 2.5 Risk of Bias Assessment

The quality of the studies was assessed by two unblinded reviewers, using different tools according to study design: the Modesti et al. adaptation of the Newcastle‐Ottawa Scale for cross‐sectional studies (Modesti et al. 2016); the Newcastle‐Ottawa Scale for cohort studies (Wells et al. 2025); the RoB 2 tool (Higgins et al. 2011), and the ROBINS‐I tool (Sterne et al. 2016). Any disagreements between reviewers were resolved through discussion and consensus. When necessary, a senior reviewer was consulted to reach a final decision. All information for the evaluation of risk of bias is presented in Appendix S2.

### 2.6 Data Synthesis and Statistical Analysis

The meta‐analysis conducted brought together studies assessing the QoL of caregivers of individuals with ASD and of the individuals themselves, encompassing multiple domains—physical, emotional, social, and overall QoL scores.

Data were extracted independently by two reviewers using a standardized spreadsheet developed for this review, capturing the following variables: study authorship, year, country, study design, population characteristics (including sample size, age, and sex/gender distribution), QoL instrument and its domains assessed, and the reported scores (means and standard deviations) and comparison group data where available. Inconsistencies in data extraction were resolved by a third senior reviewer. For studies that reported duplicate data or overlapping samples, only the most comprehensive and recent dataset was retained to avoid duplication.

All statistical analyses were conducted using R version 4.3.2 (R Foundation for Statistical Computing, Vienna, Austria) and the meta and metafor packages. The primary outcome was the pooled mean of QoL scores of individuals with ASD and their caregivers, as well as the mean differences between caregivers and controls, and between patients and their respective controls. Preplanned secondary outcomes included domain‐specific QoL scores (emotional, social, and physical) and moderator analyses based on participants' age, national HDI and continent. Studies were classified into three age‐based groups: (i) studies with samples comprising participants aged up to 10 years; (ii) studies with samples including participants older than 10 years; and (iii) studies involving both children and adolescents. Random‐effects models were used to calculate pooled mean and mean differences and 95% confidence intervals (CIs), accounting for between‐study variability. Heterogeneity was quantified using the *I* ^2^ statistic. Subgroup analyses and meta‐regressions were conducted to explore potential sources of heterogeneity, including age (age categorization was conducted considering three groups: studies involving children, studies involving adolescents, and studies presenting combined data for children and adolescents), geographic region and HDI. Publication bias was assessed through visual inspection of funnel plot and confirmed with Egger's test.

## 3 Results

This systematic review included 216 studies (Complete list of references available in Appendix S6) conducted between 2013 and 2025 across a variety of countries, of which 173 were eligible for the meta‐analysis. The studies explored the QoL of either caregivers of children with ASD or the children themselves, employing validated instruments such as the SF‐36, WHOQOL‐BREF, PedsQL, and QoLA. Figure 1 shows the PRISMA flow diagram of the study selection. Additionally, details of study exclusions are available in Appendix S3.

**FIGURE 1:**
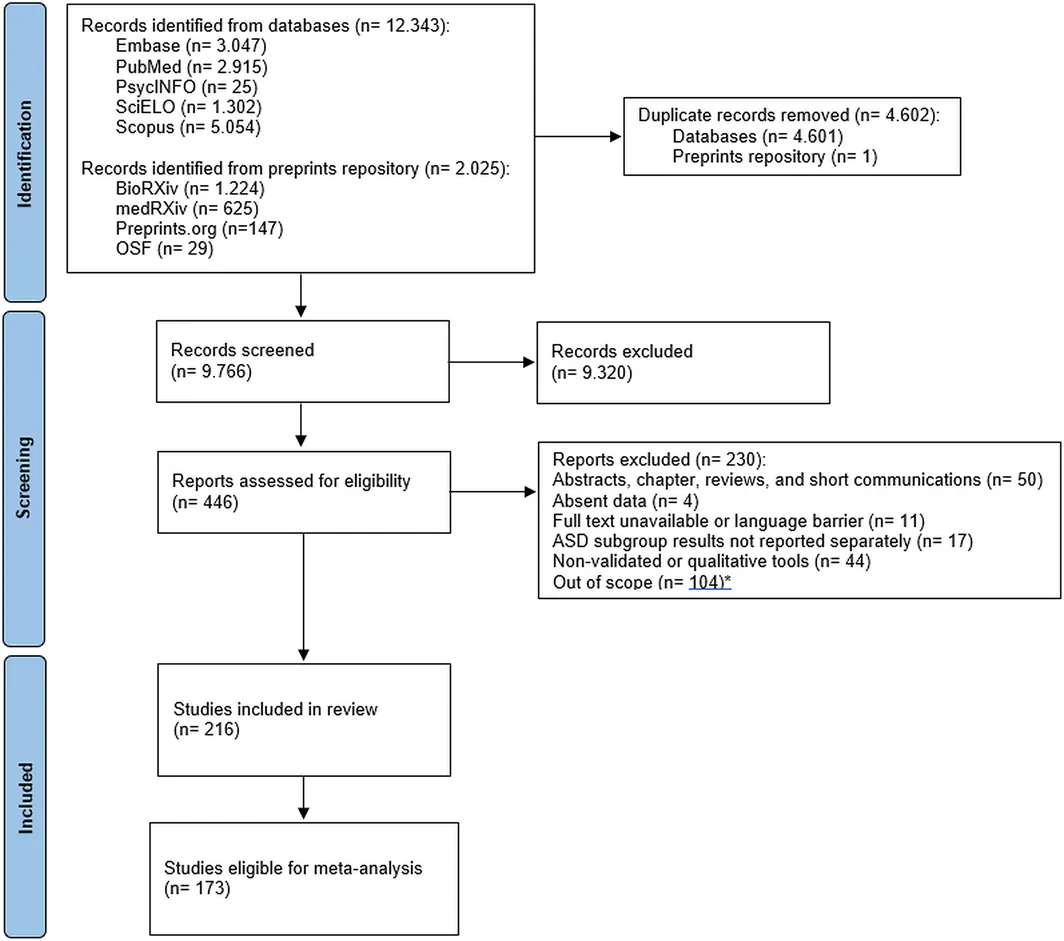
Flow diagram. ASD, autism spectrum disorder. *Out of scope = studies that did not investigate quality of life or included adult patients with ASD, professional caregivers, siblings, or teachers.

Among the studies included in the review, the majority employed a cross‐sectional design (*n* = 184). Regarding the target population, most studies focused on caregivers (*n* = 147), whereas 80 studies directly assessed children and adolescents with ASD. Of the 216 included studies, 48 included a comparison/control group, whereas the remaining studies evaluated only individuals with ASD and/or their caregivers without a control group. Only 24 studies involved some form of intervention, suggesting that most investigations have been limited to descriptive or correlational assessments (Table 1).

**TABLE 1:** Characteristics of the included studies.*

Study	Study type	Instrument	Participants	Controls	Mean quality of life
Responsables					
Barbosa et al. 2009	Cross‐sectional	WHOQOL‐BREF	150	—	21.25 ± 12.25
Lee et al. 2009	Cross‐sectional	SF‐36	89	46	76.85 ± 16.84 (ASD) 84.42 ± 10.63 (TD)
Shu 2009	Cross‐sectional	WHOQOL‐BREF	104	—	78.80 ± 14.80
Fávero‐Nunes and Santos 2010	Cross‐sectional	WHOQOL‐BREF	20	—	64.36 ± 15.26
Eskow et al. 2011	Cross‐sectional	FQoL	855	—	73.00 ± 14.20
Khanna et al. 2011	Cross‐sectional	SF‐12	304	—	44.38 ± 12.77
Roberts et al. 2011	Clinical trial	FQoL	85	—	74.00 ± 11.80
Khanna et al. 2012	Validation Study	SF‐12	304	—	—
Kheir et al. 2012	Cross‐sectional	SF‐36	56	42	45.39 ± 10.77 (ASD) 47.28 ± 9.03 (TD)
Kheir et al. 2012	Cross‐sectional	SF‐36	56	42	—
Lee et al. 2012	Cross‐sectional	SF‐36	113	47	76.80 ± 17.28 (ASD) 84.25 ± 10.56 (TD)
Rizk et al. 2012	Cross‐sectional	SF‐12	33	—	46.45 ± 11.75
Dardas and Ahmad 2013	Cross‐sectional	WHOQOL‐BREF	184	—	80.00 ± 16.00
Meral et al. 2013	Cross‐sectional	FQoL	3009	—	91.26 ± 17.57
Tekinarslan 2013	Cross‐sectional	WHOQOL‐BREF	93	78 (DS); 81 (CP)	57.36 ± 17.65 (ASD) 58.01 ± 18.19 (DS) 52.96 ± 19.88 (CP)
Werner and Shulman 2013	Cross‐sectional	PWI	56	—	61.68 ± 20.99
Chiang 2014	Clinical trial	WHOQOL‐BREF	9	—	19.90 ± 6.70
Dardas and Ahmad 2014	Validation Study	WHOQOL‐BREF	184	—	—
Dardas and Ahmad 2014	Cross‐sectional	WHOQOL‐BREF	184	—	—
Dardas and Ahmad 2014	Cross‐sectional	WHOQOL‐BREF	184	—	80.00 ± 16.00
Dardas and Ahmad 2014	Cross‐sectional	WHOQOL‐BREF	184	—	61.39 ± 12.57
Eapen et al. 2014	Validation Study	QoLA—part A	23	16	62.11 ± 15.38 (ASD) 78.57 ± 12.56 (TD)
		QoLA—part B	23	16	68.52 ± 17.56 (ASD) 94.31 ± 6.14 (TD)
		WHOQOL‐BREF	23	—	57.27 ± 13.36
Ji et al. 2014	Clinical trial	SF‐36	49	—	57.94 ± 11.87
Ji et al. 2014	Cross‐sectional	SF‐36	273	—	52.15 ± 16.13
Kuhlthau et al. 2014	Cross‐sectional	SF‐6D	224	—	74.00 ± 12.00
		EQUATION 5D	224	—	85.00 ± 14.00
		CarerQoL	224	—	40.5 ± 24.70
McStay et al. 2014	Cross‐sectional	FQoL	140	—	73.00 ± 8.60
McStay et al. 2014	Cross‐sectional	FQoL	196	—	—
Perumal et al. 2014	Cross‐sectional	WHOQOL‐BREF	54	38 (PD); 48 (TD)	63.63 ± 12.18 (ASD) 78.07 ± 6.95 (PD) 83.89 ± 3.88 (TD)
Tung et al. 2014	Cross‐sectional	WHOQOL‐BREF	82	—	—
Dardas et al. 2015	Cross‐sectional	WHOQOL‐BREF	101	—	74.17
Fourcade et al. 2015	Cross‐sectional	GHQ12	224	—	—
Gardier and Iarocci 2015	Cross‐sectional	FQoL	84	—	72.40 ± 12.60
Hoefman et al. 2015	Validation Study	EQUATION 5D	224	—	85.00 ± 14.00
Hoefman et al. 2015	Validation Study	SF‐6D	224	—	74.00 ± 12.00
Hoefman et al. 2015	Validation Study	CarerQoL	224	—	80.72 ± 12.88
Kousha et al. 2015	Cross‐sectional	WHOQOL‐BREF	127	—	20.91 ± 7.66
Lara and Los Pinos 2015	Cross‐sectional	ECVF	12	—	—
Schertz et al. 2015	Cross‐sectional	FQoL	29	—	—
Siah and Tan 2015	Cross‐sectional	WHOQOL‐BREF	96	—	54.95 ± 14.49
Forouzanfar et al. 2016	Cross‐sectional	SF‐36	203	—	48.45 ± 25.48
Rayan and Ahmad 2016	Clinical trial	WHOQOL‐BREF	104	—	51.47
Schlebusch et al. 2016	Cross‐sectional	FQoL	180	—	76.60 ± 12.20
Siah and Tan 2016	Cross‐sectional	WHOQOL‐BREF	92	—	—
Tait et al. 2016	Cross‐sectional	FQoL	75	—	—
Amirian et al. 2017	Cross‐sectional	SF‐36	97	—	54.99 ± 19.85
Due et al. 2017	Cross‐sectional	QoLA—part A	27	—	65.43 ± 9.79
Due et al. 2017	Cross‐sectional	QoLA—part B	27	—	58.70 ± 16.00
Jones et al. 2017	Cross‐sectional	FQoL	151	—	—
McAuliffe et al. 2017	Cross‐sectional	WHOQOL‐BREF	207	—	—
Nuske et al. 2017	Cross‐sectional	PedsQL‐Family Impact	43	28	52.60 ± 20.5 (ASD) 80.98 ± 14.56 (TD)
Pisula et al. 2017	Cross‐sectional	WHOQOL‐BREF	98	104	14.13 ± 2.22
Russell et al. 2017	Cross‐sectional	SF‐12	50	—	44.91 ± 17.76
Schlebusch et al. 2017	Cross‐sectional	FQoL	180	—	76.60 ± 12.20
Alhazmi et al. 2018	Cross‐sectional	WHOQOL‐BREF	26	26	50.50 ± 20.00 (ASD) 88.70 ± 13.70 (TD)
Chaabène et al. 2018	Cross‐sectional	SF‐36	50	—	52.95 ± 20.48
Derguy et al. 2018	Cross‐sectional	WHOQOL‐BREF	115	—	—
Eskow et al. 2018	Cross‐sectional	FQoL	313	—	78.20 ± 12.80
Kuru et al. 2018	Cross‐sectional	QOL‐8‐TR	90	—	65.43 ± 12.28
Lee et al. 2018	Cross‐sectional	SF‐12	132	—	—
Özgür et al. 2018	Cross‐sectional	QoLA—part A	160	—	66.94 ± 13.53
Poirier et al. 2018	Cross‐sectional	QOLS	30	—	43.99 ± 13.10
Willet et al. 2018	Cross‐sectional	QoLA—part A	156	—	60.77 ± 12.20
Ying et al. 2018	Cross‐sectional	SF‐36	406	513	—
Deshpande et al. 2019	Clinical trial	SF‐36	48	—	—
Jain et al. 2019	Cross‐sectional	WHOQOL‐BREF	90	—	44.40 ± 18.60
Kousha et al. 2019	Cross‐sectional	SF‐36	90	90	53.61 ± 2.92 (ASD) 58.86 ± 2.68 (TD)
Mello et al. 2019	Cross‐sectional	FQoL	493	—	73.60 ± 11.60
Millau et al. 2019	Cross‐sectional	FQoL	104	—	74.20 ± 11.00
Naheed et al. 2019	Cross‐sectional	EQ‐5D	388	—	67.00 ± 32.00
Roberts et al. 2019	Clinical trial	PedsQL‐Family Impact	23	—	44.53 ± 14.98
Zeng et al. 2019	Cross‐sectional	FQoL	226	—	—
Adams et al. 2020	Cross‐sectional	WHOQOL‐BREF	64	—	72.00 ± 12.00
Alenazi et al. 2020	Cross‐sectional	SF‐36	84	—	54.30 ± 30.29
Garrido et al. 2020	Cross‐sectional	FQoL	24	25	61.17 ± 11.69 (ASD) 75.31 ± 5.97 (TD)
Likhitweeranwong et al. 2020	Cross‐sectional	WHOQOL‐BREF	61	63	79.10 ± 12.18 (ASD) 82.10 ± 9.25 (TD)
Moretto et al. 2020	Cross‐sectional	WHOQOL‐BREF	41	—	57.65 ± 22.45
Öz et al. 2020	Cross‐sectional	QoLA—part A	69	—	64.33 ± 12.56
		QoLA—part B	69	—	68.16 ± 17.60
Parsons et al. 2020	Cross‐sectional	WHOQOL‐BREF	278	—	58.69 ± 16.20
Renford et al. 2020	Cross‐sectional	WHOQOL‐BREF	120	—	—
Selvakumar and Panicker 2020	Cross‐sectional	WHOQOL‐BREF	30	—	70.3
ten Hoopen et al. 2020	Cross‐sectional	EQ‐5D	81	—	84.00 ± 17.00
		CarerQoL	81	—	77.33 ± 16.44
Waheed et al. 2020	Cross‐sectional	WHOQOL‐BREF	50	—	53.36 ± 9.36
Wang et al. 2020	Cross‐sectional	FQoL	420	—	—
Zeng et al. 2020	Cross‐sectional	FQoL	438	—	—
Akhani et al. 2021	Clinical trial	WHOQOL‐BREF	44	—	82.96 ± 9.77
Alhuzimi 2021	Cross‐sectional	GHQ12	150	—	71.00 ± 9.60
Asahar et al. 2021	Cross‐sectional	QoLA—part A	116	—	63.25 ± 12.32
		QoLA—part B	116	—	56.55 ± 12.35
Cappe et al. 2021	Clinical trial	EQVPTSA	35	—	43.16 ± 14.70
Celik et al. 2021	Cross‐sectional	QoLA—part A	49	—	72.95 ± 5.41
		QoLA—part B	49	—	66.36 ± 4.57
Chezan et al. 2021	Validation Study	QOLASD‐C	309	—	—
Gabra and Hashem 2021	Cross‐sectional	WHOQOL‐BREF	38	34 (ADHD)	59.84 ± 14.08 (ASD) 70.05 ± 13.04 (ADHD)
Gabra et al. 2021	Cross‐sectional	WHOQOL‐BREF	70	—	—
Green et al. 2021	Cross‐sectional	WEWMBS	121	—	64.57 ± 12.24
Hand et al. 2021	Cross‐sectional	SF‐12	367	—	47.61 ± 44.41
Leader et al. 2021	Cross‐sectional	WHOQOL‐BREF	12	—	59.81 ± 13.72
Martin et al. 2021	Cross‐sectional	AQoL‐4D	234	—	69.00 ± 22.00
Pecor et al. 2021	Cross‐sectional	PedsQL‐Family Impact	170	91 (TD); 152 (ADHD); 167 (ADHD + ASD)	—
Pratesi et al. 2021	Validation Study	ASDPC‐QoL	881	—	46.30 ± 10.35
Sorrenti et al. 2021	Cross‐sectional	QoLA	84	88	40.39 ± 6.13 (ASD) 46.45 ± 7.42 (TD)
Taylor et al. 2021	Clinical trial	WEWMBS	248	—	70.37 ± 13.5
ten Hoopen et al. 2021	Cross‐sectional	EQUATION 5D	81	—	—
		CarerQoL	81	—	—
Vaz et al. 2021	Cross‐sectional	WHOQOL‐BREF	278	—	—
Wang et al. 2021	Cross‐sectional	FQoL	79	—	71.30 ± 12.31
Zhao et al. 2021	Clinical trial	WHOQOL‐BREF	75	—	40.48 ± 2.78
ABD‐Alnabi and Sachet 2022	Cross‐sectional	WHOQOL‐BREF	120	—	57.69 ± 24.95
ALBusaidi et al. 2022	Cross‐sectional	WHOQOL‐BREF	304	—	65.02 ± 8.29
Al‐Farsi et al. 2022	Cross‐sectional	WHOQOL‐BREF	220	125 (TD); 109 (ID)	64.75 ± 16.87 (ASD) 75.75 ± 14.97 (TD) 72.17 ± 15.47 (ID)
Al‐Masa'deh et al. 2022	Clinical trial	QOLS	30	—	81.33 ± 11.80
Beheshti et al. 2022	Cross‐sectional	QoLA	88	—	—
Cerit et al. 2022	Clinical trial	QoLA—part A	15	—	62.79 ± 7.14
		QoLA—part B	15	—	56.00 ± 4.04
De Castro et al. 2022	Cross‐sectional	WHOQOL‐BREF	77	—	61.30 ± 20.10
Drüsedau et al. 2022	Clinical trial	ILK	25	—	62.14 ± 11.21
Eapen et al. 2022	Cross‐sectional	QoLA—part A	97	—	70.26 ± 15.48
		QoLA—part B	97	—	71.58 ± 18.90
Feng et al. 2022	Cross‐sectional	FQoL	260	—	63.14 ± 11.76
Green et al. 2022	Clinical trial	WEWMBS	117	117	70.35 ± 13.49
Herrera et al. 2022	Cross‐sectional	ECVF	96	—	77.80 ± 9.20
Ismail et al. 2022	Cross‐sectional	QoLA	260	—	—
Lanyi et al. 2022	Cross‐sectional	WHOQOL‐BREF	152	—	53.99 ± 22.21
Lei and Kantor 2022	Cross‐sectional	FQoL	163	—	68.00 ± 12.40
Logrieco et al. 2022	Cross‐sectional	PedsQL‐Family Impact	211	—	52.00 ± 19.20
Losada‐Puente et al. 2022	Cross‐sectional	FQoL	45	32 (other disabilities)	57.40 ± 25.20 (ASD) 81.20 ± 15.20 (other disabilities)
Naheed et al. 2022	Clinical trial	EQ‐5D	160	—	76.00 ± 23.00
Patel et al. 2022	Cross‐sectional	WHOQOL‐BREF	40	—	44.09 ± 15.58
Romaniuk et al. 2022	Cross‐sectional	FQoL	46	22 (ADHD); 49 (ADHD+ASD)	77.00 ± 7.80 (ASD) 76.80 ± 9.00 (ADHD) 73.20 ± 15.20 (ADHD + ASD)
Vahedparast et al. 2022	Cross‐sectional	SF‐36	192	—	—
Ahmed et al. 2023	Cross‐sectional	WHOQOL‐BREF	201	—	46.40 ± 12.00
Eapen et al. 2023	Cross‐sectional	QoLA—part A	1121	—	67.54 ± 14.60
		QoLA—part B	1121	—	55.18 ± 18.63
Fayyz et al. 2023	Cross‐sectional	QoLA—part A	330	—	53.60 ± 19.16
		QoLA—part B	330	—	62.04 ± 17.04
Howe et al. 2023	Cross‐sectional	FQoL	26	—	72.54 ± 10.39
Lee et al. 2023	Cross‐sectional	CareQoL‐7D	20	—	—
Lei and Kantor 2023	Cross‐sectional	FQoL	165	—	68.00 ± 12.40
Mannion and Leader 2023	Cross‐sectional	WHOQOL‐BREF	409	—	60.45 ± 20.67
Masuri et al. 2023	Cross‐sectional	WHOQOL‐BREF	73	—	59.24 ± 13.05
Papadopoulos et al. 2023	Cross‐sectional	FQoL	53	—	73.60 ± 11.80
Raju et al. 2023	Cross‐sectional	WHOQOL‐BREF	30	30	58.77 ± 19.57 (ASD) 76.16 ± 11.29 (TD)
Ran et al. 2023	Cross‐sectional	SF‐12	6120	—	38.65 ± 5.77
Robeson et al. 2023	Cohort	WHO‐5	52	—	—
Russell et al. 2023	Cross‐sectional	SF‐12	62	—	48.40 ± 9.15
		FQoL	62	—	78.40 ± 12.20
Soccorso et al. 2023	Cross‐sectional	PedsQL‐Family Impact	177	—	69.12 ± 19.87
AlHefdhi et al. 2024	Cross‐sectional	WHOQOL‐BREF	99	—	72.93 ± 4.30
Cardelle‐Perez et al. 2024	Cross‐sectional	ECVF	23	—	52.49 ± 17.83
Clark and Dissanayake 2024	Cohort	QoLA—part A	73	—	68.93 ± 13.05
		QoLA—part B	73	—	64.94 ± 21.86
Dai et al. 2024	Cohort	FQoL	156	—	70.20 ± 16.60
Davy et al. 2024	Cross‐sectional	QoLA	151	—	69.17 ± 12.31
Durhan et al. 2024	Cross‐sectional	QoLA	52	—	68.83 ± 10;58
Musetti et al. 2024	Cross‐sectional	QoLA—part A	188	—	59.64 ± 15.30
		QoLA—part B	188	—	62.87 ± 19.66
Palmer et al. 2024	Clinical trial	QoLA—part A	41	—	69.34 ± 2.70
		QoLA—part B	41	—	60.07 ± 3.67
Salami and Alhalal 2024	Cross‐sectional	WHOQOL‐BREF	376	—	61.25 ± 23.29
Ye et al. 2024	Cross‐sectional	FQoL	237	—	68.60 ± 12.20
ASD children and adolescents					
Elias et al. 2006	Cross‐sectional	AUQEI	20	20	—
Kuhlthau et al. 2010	Cross‐sectional	PedsQL	286	—	65.2 ± 15.9
Shipman et al. 2011	Validation Study	PedsQL	39	—	—
Sheldrick et al. 2012	Cross‐sectional	PedsQL	39	—	—
Sikora et al. 2012	Cross‐sectional	PedsQL	2202	—	68.90 ± 46.46 (ASD) 59.10 ± 47.86 (ASD and ADHD)
Tavernor et al. 2012	Cross‐sectional	PedsQL	10	—	—
		KIDSCREEN‐27			—
Tilford et al. 2012	Cross‐sectional	HUI‐3	150	—	66.00 ± 23.00
		QWB‐SA			59.00 ± 16.00
Varni et al. 2012	Clinical trial	PedsQL	243	—	41.5 ± 15.58
Kose et al. 2013	Cross‐sectional	PedsQL	102	—	56.80 ± 14.40
Kuhlthau et al. 2013	Cross‐sectional	CHIP‐CE	71	—	37.67 ± 10.98
Chuang et al. 2014	Cross‐sectional	TAPQOL‐C	106	—	—
Delahaye et al. 2014	Cross‐sectional	PedsQL	86	—	64.18 ± 15.64
Domellöf et al. 2014	Cross‐sectional	EQ‐5D‐Y	61	—	—
Kose et al. 2013	Cross‐sectional	PedsQL	102	—	56.80 ± 14.40
Kuhlthau et al. 2013	Cross‐sectional	CHIP‐CE	71	—	37.67 ± 10.98
Lanning et al. 2014	Clinical trial	PedsQL	10	—	50.50 ± 23.62 (social) 61.1 ± 28.65 (physical)
McStay et al. 2014	Cross‐sectional	PedsQL	150	—	64.12 ± 11.88
Clark et al. 2015	Cross‐sectional	KIDSCREEN‐52	22	—	40.7 ± 11.88
Herpers et al. 2015	Cross‐sectional	KIDSCREEN‐27	437	—	66.59
Hoefman et al. 2015	Validation Study	QWB‐SA	224	—	59.0 ± 16.0
Jahan et al. 2015	Cross‐sectional	PedsQL	149	—	71.38 ± 16.76
Jamison and Shuttler 2015	Cross‐sectional	YQOL‐R	12	22	61.65 ± 16.0 (ASD) 93.64 ± 8.6 (TD)
Potvin et al. 2015	Cross‐sectional	PedsQL	30	31	56.1 ± 13.58 (ASD) 85.54 ± 8.75 (TD)
Randall et al. 2015	Cross‐sectional	PedsQL	161	8201	59.13 ± 16.02 (ASD) 78.29 ± 13.34 (TD)
Sarol and Çimen 2015	Clinical trial	PedsQL	59	—	49.75 ± 12.75
van Steensel and Bögels 2015	Clinical trial	EQ‐5D	79	95 (Anxiety Disorders)	—
Vries et al. 2015	Cross‐sectional	PedsQL	119	73	32.9 ± 11.6 (ASD) 9.2 ± 7.1 (TD)
Biscaldi‐Schäfer et al. 2016	Cross‐sectional	ILK	38	—	17.21 ± 3.06
Broquere et al. 2016	Cross‐sectional	KIDSCREEN‐27	26	20 (ASD + Anxiety)	—
Egilson et al. 2016	Cross‐sectional	KIDSCREEN‐27	96	—	37.09 ± 12.73 (social) 40.85 ± 9.33 (physical)
Öztürk et al. 2016	Cross‐sectional	PedsQL	102	40 (ADHD); 44 (Asthma); 39 (TD)	—
Flor et al. 2017	Cross‐sectional	PedsQL	808 (Essential ASD)	55 (Complex ASD)	64.7 ± 15.8 (Essential ASD) 60.4 ± 17.7 (Complex ASD)
Hatfield et al. 2017	Clinical trial	PWI‐SC	94	—	62.06 ± 21.03
Kuhlthau et al. 2017	Cohort	PedsQL	4910	—	65.00 ± 15.80
Toscano et al. 2017	Clinical trial	PedsQL	64	—	32.55 ± 15.67
Vries et al. 2017	Clinical trial	PedsQL	101	—	—
Gengoux et al. 2018	Clinical trial	PedsQL	22	—	64.15 ± 18.11
Hedgecock et al. 2018	Cross‐sectional	PedsQL	3155	—	68.82 ± 14.78
Adams et al. 2019	Cohort	PedsQL	71	—	58.38 ± 22.1
Coales et al. 2019	Cross‐sectional	KIDSCREEN‐52	51	63 (DLD)	42.66 ± 10.25 (ASD) 47.82 ± 12.13 (DLD)
Günal et al. 2019	Cross‐sectional	CHQ	32	30	21.53 ± 10.56 (ASD) 25.0 ± 11.6 (TD)
Güneş and Ekinci 2019	Cross‐sectional	KINDL	81	79	84.8 ± 15.7 (ASD) 89.8 ± 13.1 (TD)
Kilincaslan et al. 2019	Cross‐sectional	PedsQL	51	51 (ID)	56.62 ± 11.47 (ASD) 56.62 ± 13.26 (ID)
Mazon et al. 2019	Cross‐sectional	AuQuEI	27	18 (ID)	61.77 ± 13.04 (ASD) 62.78 ± 11.39 (ID)
Adams et al. 2020	Cross‐sectional	PedsQL	64	—	50.8 ± 15.6
Anixt et al. 2020	Cross‐sectional	PGH‐7	526	—	67.43 ± 10.57
Bent et al. 2020	Cohort	PedsQL	29	—	—
Katsiana et al. 2020	Cross‐sectional	PedsQL	61	55 (DS); 90 (TD)	65.68 ± 17.57 (ASD) 69.98 ± 14.03 (DS) 88.68 ± 11.49 (TD)
Lal D V et al. 2020	Cross‐sectional	PedsQL	98	100	49.27 ± 24.3
Likhitweeranwong et al. 2020	Cross‐sectional	PedsQL	61	63	56.49 ± 13.8 (ASD) 70.96 ± 18.45 (TD)
May et al. 2020	Cross‐sectional	PedsQL	240	—	61.13 ± 16.35
Oakley et al. 2020	Cross‐sectional	CHIP‐CE	238	143	—
ten Hoopen et al. 2020	Cross‐sectional	EQ‐5D	88	—	67.00 ± 26.00
Williams et al. 2020	Cross‐sectional	QI‐Disability	133	151 (CP); 89 (DS); 62 (RS)	68.40 ± 11.00 (ASD) 66.6 ± 13.5 (CP) 77.6 ± 11.7 (DS) 65.8 ± 11.8 (RS)
Chezan et al. 2021	Validation Study	QOLASD‐C	233	—	—
		PedsQL	233	—	—
Leader et al. 2021	Cross‐sectional	PedsQL	133	—	18.79 ± 5.80
Leader et al. 2021	Cross‐sectional	PedsQL	120	—	47.91 ± 17.04
Lopez‐Espejo et al. 2021	Cross‐sectional	PedsQL	93	—	75.6 ± 8.37
Menezes and Mazurek 2021	Cross‐sectional	PedsQL	470	—	67.28 ± 20.95
Pavlova et al. 2021	Cross‐sectional	PedsQL	18	30	50.18 ± 15.1 (ASD) 78.1 ± 15.55 (TD)
Drüsedau et al. 2022	Clinical trial	ILK	25	—	72.86 ± 11.43
Green et al. 2022	Clinical trial	CHU9D	230	—	37.89 ± 11.89
Kim et al. 2022	Clinical trial	PedsQL	24	—	49.88 ± 14.32
Leader et al. 2022	Cross‐sectional	PedsQL	51	67 (ASD and ADHD)	65.06 ± 12.06 (ASD) 46.84 ± 16 (ASD and ADHD)
Leonard et al. 2022	Cross‐sectional	QI‐Disability	130	—	68.03 ± 10.9
Logrieco et al. 2022	Cross‐sectional	PedsQL	194	—	61.1 ± 16.8
		PedsQL	31	—	—
Saigh and Bagadood 2022	Cross‐sectional	KIDSCREEN‐52	51	341	—
Sari et al. 2022	Cross‐sectional	PedsQL	65	70	45.97 ± 10.94 (ASD) 71.07 ± 12.55 (TD)
		KIDSCREEN‐27			26.60 ± 16.80 (ASD) 53.40 ± 22.40 (TD)
Schneider et al. 2022	Cohort	KIDSCREEN‐27	32	—	61.22 ± 27.62
Süzen et al. 2022	Cross‐sectional	PedsQL	28	—	49.87 ± 19.08
Albadry et al. 2023	Cross‐sectional	PedsQL	60	—	29.61 ± 3.79
Bulkley et al. 2023	Cross‐sectional	PedsQL	564	468 (Asthma); 429 (TD)	63.8 ± 20.59 (ASD) 79.75 ± 17.69 (Asthma) 83.5 ± 16.24 (TD)
Chezan et al. 2023	Validation Study	QOLASD‐C	1317	—	—
Howe et al. 2023	Cross‐sectional	PedsQL	20	—	50.6 ± 15.73
Lichtlé et al. 2023	Validation Study	PedsQL	157	—	58.51 ± 15.14
Lopes‐Espejo et al. 2023	Cross‐sectional	PedsQL	131	—	69.5 ± 15.33
Kinter et al. 2024	Cross‐sectional	PedsQL	37	37 (ASD + ARFID); 37 (TD)	—
Mahjoob et al. 2024	Cross‐sectional	KINDL	273	135 (ADHD); 38 (OCD); 7 (SubADHD); 1 (SubOCD); 161 (TD)	63.75 ± 12.26 (ASD) 64.04 ± 13.16 (ADHD) 64.14 ± 12.82 (OCD) 68.01 ± 9.04 (SubADHD) 87.5 (SubOCD) 72.16 ± 11.15 (TD)
Prasitwut et al. 2024	Clinical trial	PedsQL	23	—	35.32 ± 15
Stiller et al. 2024	Cross‐sectional	ILK	282	—	67.00 ± 11.00

Abbreviations: ADHD, attention deficit hyperactivity disorder; AQoL‐4D, assessment of quality of life; ASD, autism spectrum disorder; ASDPC‐QoL, autistic spectrum disorder parent/caregiver quality of life; AUQUEI, Autoquestionnaire Qualité de Vie Enfant Imagé; CarerQoL, care‐related quality of life instrument; CHIP‐CE, child health and illness profile–child edition; CHQ, child health questionnaire; CHU9D, child health utility 9D; CP, cerebral palsy; DLD, developmental language disorders; DS, Down syndrome; ECVF, Escala de Calidad de Vida Familiar; EQ‐5D, EuroQol 5‐dimensions; EQVPTSA, quality of life scale for parents of a child with ASD; FQoL, Beach Center Family Quality of Life Scale; GHQ‐12, general health questionnaire‐12; HUI‐3, health utilities index mark III; ID, intellectual disability; ILK, Inventar Lebensqualität Kindern und Jugendlichen; KIDSCREEN‐27, KIDSCREEN‐27 quality of life questionnaire; KIDSCREEN‐52, KIDSCREEN‐52 quality of life questionnaire; KINDL, children's quality of life questionnaire (Kinder Lebensqualität Fragebogen); PedsQL, pediatric quality of life inventory; PGH‐7, patient‐reported outcomes measurement information system global health measure; PWI, personal wellbeing index; PWI‐SC, personal wellbeing index–school children; QI‐Disability, quality of life inventory‐disability; EUROHIS QOL‐8‐TR, quality of life index EUROHIS‐QOL‐8; QoLA, quality of life in autism; QOLASD‐C, quality of life for children with autism spectrum disorder; QOLS, quality of life scale; QWB‐SA, quality of well‐being scale–self‐administered; RS, Rett syndrome; SF‐12, 12‐item short form health survey; SF‐36, 36‐item short form health survey; SF‐6D, short form 6 dimensions; TAPQOL‐C, TNO‐AZL Preschool Children Quality of Life Questionnaire; TD, typical development; WEMWBS, Warwick–Edinburgh mental wellbeing scale; WHO‐5, World Health Organization‐five well‐being index; WHOQOL‐BREF, World Health Organization quality of life‐BREF.

* Complete references for the studies presented in Table 1 are provided in Appendix S6.

### 3.1 Meta Analysis About Patients QoL

Although 173 studies were eligible for quantitative synthesis, separate meta‐analyses were conducted according to the outcome of interest and the availability of comparable data. The pooled analysis of overall QoL in individuals with ASD included 57 studies, as only these provided sufficient data (means, standard deviations, and compatible outcome measures) for this specific analysis. The estimated mean QoL was 55.75 (95% CI: 53.26–58.35), with high heterogeneity (*I* ^2^ = 99.2%) (Figure 2A). When compared with controls, patients with ASD presented significantly lower QoL (95% CI: −26.9; −16.52, *p* < 0.001) (Figure 2C).

**FIGURE 2:**
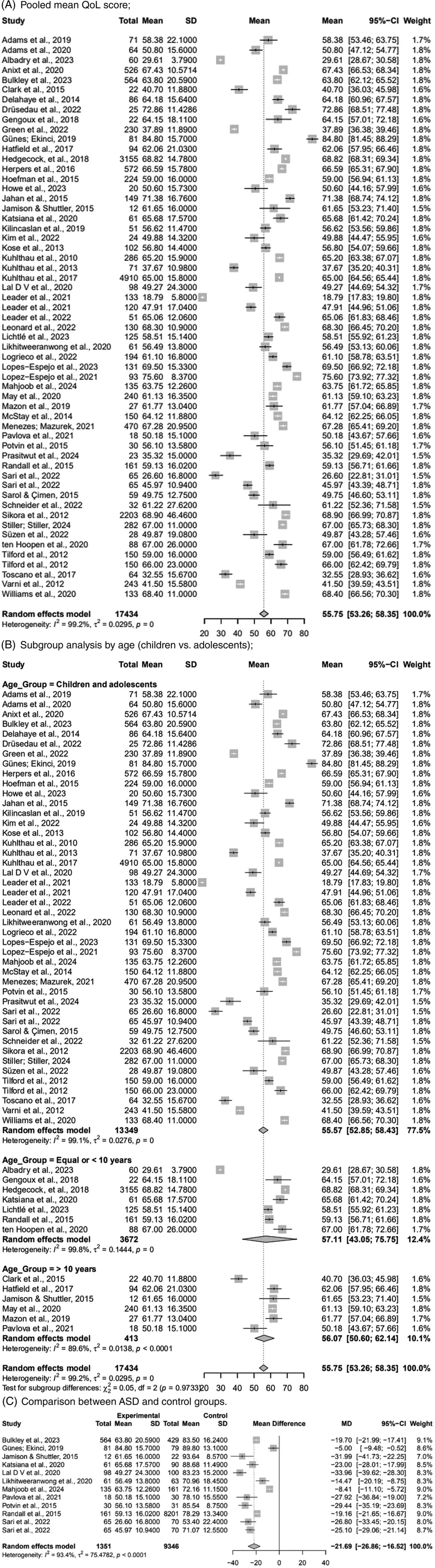
Meta‐analytic estimates of quality of life (QoL) in individuals with autism spectrum disorder (ASD). (A) Pooled mean QoL score. (B) Subgroup analysis by age (children vs. adolescents). (C) Comparison between ASD and control groups.

The meta‐analysis of mean QoL scores among caregivers of individuals with ASD revealed a generalized impairment when compared to control groups, with an overall effect size of −13.64 points (95% CI: −19.21; −8.06, *p* < 0.0001) (Figure 3A). In total, the analysis included studies with a combined sample of over 28,000 participants.

**FIGURE 3:**
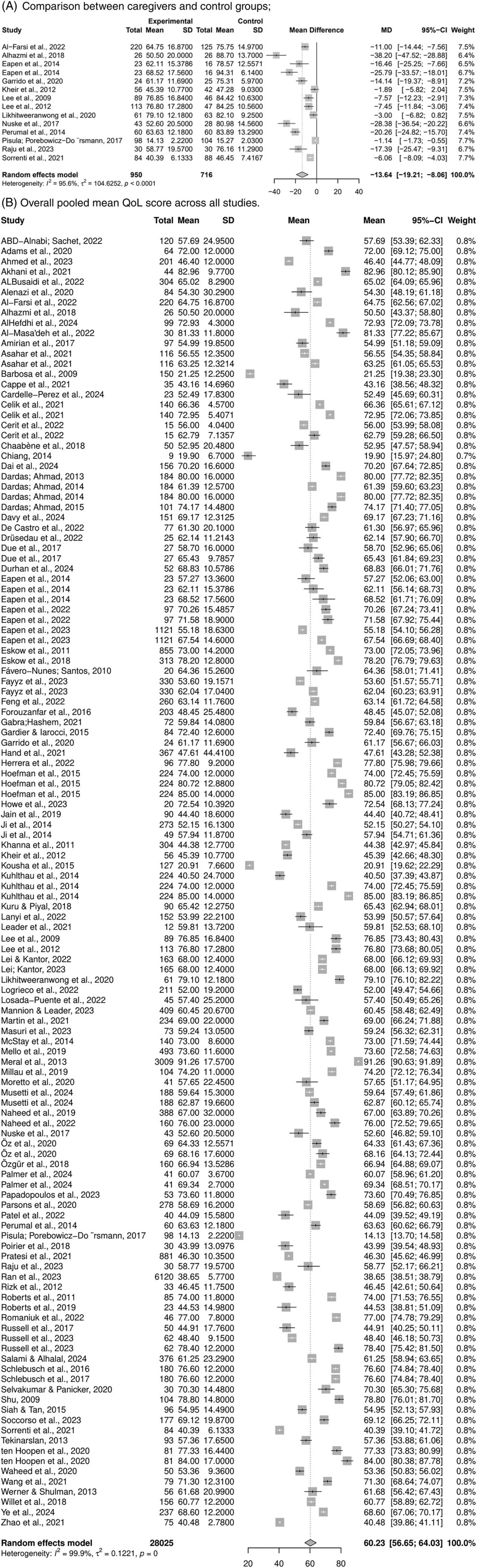
Summary of meta‐analyses assessing quality of life (QoL) among caregivers of individuals with autism spectrum disorder (ASD). (A) Comparison between caregivers and control groups. (B) Overall pooled mean QoL score across all studies.

A subgroup meta‐analysis was performed to assess whether age group influenced the mean QoL of individuals with ASD and no significant difference was found between children and adolescents (*p* = 0.9733), although children tended to show lower QoL scores (Figure 2B). Despite the observed variability—potentially attributable to methodological differences, sociocultural contexts, or sample characteristics—the overall mean QoL score was approximately 60.23 points (95% CI: 56.65–64.03) (Figure 3B).

### 3.2 Emotional Domain

Meta‐analytic data indicate that the emotional domain is one of the most affected areas in both caregivers and individuals with ASD. A total of 29 studies involving 7670 caregivers and 23 studies including 4734 individuals with ASD were analyzed to assess emotional QoL (QoL). Among caregivers, the pooled mean emotional QoL score was approximately 56.3 points (95% CI: 41.54–76.32, Appendix S4a) reflecting a marked emotional burden associated with caregiving. Similarly, individuals with ASD showed significant impairments in emotional QoL, with an overall mean score of 58.9 points (95% CI: 55.85–62.31, Appendix S4b), and several studies reporting mean values below 50—highlighting substantial emotional difficulties in this population. Age subgroup analysis for patients with ASD did not reveal statistically significant differences (*p* = 0.2211, Appendix S4c).

### 3.3 Social Domain

Meta‐analytic findings also highlight significant impairments in the social domain for both caregivers and individuals with ASD. A total of 45 studies comprising 8241 caregivers and 27 studies including 5002 individuals with ASD were analyzed. Among caregivers, the pooled mean social QoL score was 49.82 points (95% CI: 40.85–60.75, *I* ^2^ = 99.99, Appendix S4d), indicating substantial social limitations and reduced participation in social activities. Similarly, individuals with ASD had a mean score of 50.69 points (95% CI: 47.55–54.03, *I* ^2^ = 95.02, Appendix S4e), reflecting persistent social challenges that extend beyond childhood. Age subgroup analysis for individuals with ASD revealed no statistically significant differences across age groups (*p* = 0.5057, Appendix S4f).

### 3.4 Physical Domain

Analysis of the physical domain indicated moderately reduced QoL scores, with considerable variability across the included studies. A total of 61 studies involving 11,481 caregivers were analyzed and the pooled mean physical QoL score was 58.38 points (95% CI: 49.77–68.48, *I* ^2^ = 99.99, Appendix S4g). For individuals with ASD, 31 studies including 6050 individuals with ASD were analyzed and the overall mean physical QoL score was approximately 66.0 points (95% CI: 62.17–70.06, *I* ^2^ = 98.2, Appendix S4h).

### 3.5 Analysis Per Continent

Subgroup analysis was conducted to identify if there were any differences among the data observed through continents. Significant variations in mean scores across continents were observed for both QoL assessments of individuals with ASD and caregivers. The highest mean scores for patients were reported in Asia (mean = 61.68; 95% CI: 58.43–65.12) and Africa (mean = 59.38; 95% CI: 57.14–61.70), whereas the lowest were found in South America (mean = 51.13; 95% CI: 44.28–59.04) and Australia (mean = 54.69; 95% CI: 46.38–64.50). Europe (mean = 55.75; 95%) and North America (mean = 56.24; 95%) showed intermediate values. These findings suggest regional differences in functional outcomes and perceived QoL among individuals with ASD, potentially influenced by socioeconomic factors, access to services, and local public policies.

Regarding caregivers, significant differences between continents were also observed. The highest scores were reported in South America (mean = 65.60; 95% CI: 62.91–68.41) and Europe (mean = 63.58; 95% CI: 56.99–70.95), while the lowest mean was found in North America (mean = 47.28; 95% CI: 38.77–57.67). Africa (mean = 60.23; 95%), Asia (mean = 56.62; 95%), and Australia (mean = 61.14; 95%) reported intermediate levels. These findings are illustrated in the Figure provided in Appendix S5.

### 3.6 Publication Bias

Visual inspection of the funnel plots indicated asymmetry across several meta‐analytic models, suggesting the presence of small‐study effects, particularly in analyses of caregiver QoL and emotional and social domains. Egger's test confirmed significant asymmetry in these outcomes, indicating a tendency for smaller studies to report greater QoL impairments. Although some outcomes—such as the physical domain—showed minimal evidence of bias, the overall pattern suggests that publication bias is present and may modestly influence effect size estimates. Nonetheless, the consistency of findings across large samples and multiple domains suggests that the observed reductions in QoL among individuals with ASD and their caregivers are robust, even when considering potential bias.

### 3.7 Risk of Bias

Overall, the risk of bias assessment revealed substantial variability in methodological quality across studies. Longitudinal studies demonstrated moderate risk of bias, with scores ranging from 3 to 5 on the Newcastle–Ottawa Scale. Cross‐sectional studies showed the widest variation (scores 1–9), though most fell within a moderate range, indicating common limitations in sampling and confounding control. Among randomized controlled trials assessed with the ROB‐2 tool, most were rated as having “some concerns,” with only a few classified as low risk and two as high risk. Non‐randomized studies, evaluated using ROBINS‐I, were predominantly judged to have moderate to serious risk of bias. Collectively, these findings highlight that, although the evidence base is extensive, methodological weaknesses are frequent and should be considered when interpreting the results (Appendix S2).

## 4 Discussion

The findings of this systematic review and meta‐analysis confirm that both caregivers of individuals with ASD and the individuals themselves experience significantly reduced QoL across multiple domains, particularly in emotional, social, and overall functioning. These findings are consistent with previous research highlighting the substantial burden associated with ASD—both for individuals with the condition and for their caregivers—as well as the complex, lifelong support needs of this population (Vohra et al. 2014; Tedla et al. 2024).

Recently, papers demonstrated individuals with ASD score significantly below typically developing peers across general and health‐related QoL measures (Tedla et al. 2024; Thiel et al. 2024). Together, these findings reinforce a growing consensus that QoL impairment is a persistent feature of ASD, regardless of age or cultural background. Interestingly, the absence of significant differences between children and adolescents in our subgroup analysis suggests that QoL deficits may be stable throughout development. This is consistent with findings indicating that, without targeted interventions, the psychosocial challenges associated with ASD remain relatively constant over time (Van Heijst and Geurts 2015).

Although these factors were not directly evaluated in the present meta‐analysis, previous studies suggest that several interrelated mechanisms may contribute to the lower QoL observed among individuals with ASD. These include psychiatric comorbidities, barriers to social participation, and other psychosocial factors (Mason et al. 2018). These conditions exacerbate difficulties in coping, self‐perception, and daily functioning, explaining part of the emotional and social burden observed in our analysis (Egilson et al. 2017). Additionally, social participation barriers likely play a central role. Impaired social communication, heightened sensitivity to social rejection, and the stress associated with masking or camouflaging autistic traits can limit social engagement and satisfaction.

Beyond the challenges faced by individuals with ASD themselves, considerable attention has also been directed toward understanding the experiences and well‐being of their caregivers. One of the most critical implications of these findings is the high prevalence of mental health disorders among caregivers. Numerous studies have documented elevated rates of depression, anxiety, and chronic stress in parents of children with ASD (Hayes and Watson 2013; Ng et al. 2021; Chua et al. 2023). A study demonstrated that caring for children with Asperger syndrome is associated with poorer self‐reported health and greater medication use among parents, highlighting the prolonged psychological and physiological toll of caregiving, even in the absence of marked biological stress responses (Ruiz‐Robledillo and Moya‐Albiol 2013). These mental health challenges are often exacerbated by a lack of formal support systems, inadequate respite care, and social isolation (Zuckerman et al. 2015).

Empirical research further highlights the pivotal role of social support in mitigating the adverse effects of caregiving on mental health. Caregivers with access to robust support networks—comprising family, friends, and professional resources—tend to report lower stress levels and improved QoL (Robinson and Weiss 2020). Moreover, coping strategies such as mindfulness‐based interventions, cognitive‐behavioral therapy (CBT), and structured respite care have been shown to promote better mental well‐being among caregivers (Dardas and Ahmad 2015). Nonetheless, many still face significant barriers in accessing these support systems, struggling to reconcile caregiving duties with personal and professional obligations.

The reduced QoL observed in this study can be attributed to multiple intersecting stressors. Parental overload is a key component—defined not only by the number of daily caregiving tasks but also by their emotional intensity and unpredictability. Caregivers frequently report disrupted sleep patterns, reduced participation in social and professional activities, and chronic fatigue (Estes et al. 2009). These stressors accumulate over time, resulting in what is often referred to as “caregiver burnout,” a syndrome with both physical and psychological consequences (Kuhlthau et al. 2015).

Importantly, while caregiving demands are highest in early childhood due to intensive behavioral needs and therapy schedules, they do not necessarily diminish with time. Rather, they often shift in nature. As individuals with ASD grow older, families face new challenges related to education, social integration, employment, and independent living (Shattuck et al. 2011). This life‐span perspective reveals that the caregiving burden persists in adolescence and adulthood, particularly in contexts where adult services are limited or inaccessible (Anderson et al. 2018). Moreover, the lack of appropriate transition services between pediatric and adult care systems contributes to uncertainty and continued stress for both individuals with ASD and their caregivers.

For individuals with ASD, interventions focusing on communication, adaptive functioning, and social skills are essential in promoting autonomy and reducing long‐term care needs (Lord et al. 2022). Early behavioral therapies and naturalistic developmental behavioral interventions have shown long‐term benefits in cognitive and social development (Zwaigenbaum et al. 2015). However, access to these services remains uneven across regions and socio‐economic groups, as reflected in this study's finding of a positive association between QoL scores and national HDI. These findings should not be interpreted as evidence of causal relationships. Instead, they likely reflect complex contextual differences across countries and regions, including variations in healthcare systems, service availability, socioeconomic conditions, cultural perceptions of disability, and support policies.

Additionally, the consistent deficits observed in emotional QoL for both caregivers and individuals with ASD emphasize the need for targeted interventions in mental health. Emotional distress, including feelings of hopelessness, isolation, and chronic worry, emerged as a core concern across studies. Yet, many caregivers report limited access to psychological services, particularly those tailored to ASD‐related challenges (Carbone et al. 2010).

Regarding the continent‐based analyses, a comparison between the two datasets reveals contrasting patterns across regions. For instance, North America presents one of the highest mean scores related to individuals with ASD, yet the lowest perceived caregiver QoL. This may reflect greater effectiveness of direct services aimed at the individual with ASD, which do not necessarily extend to family care or caregiver support. This pattern may also suggest that intensive service models, while benefiting individuals with ASD, can place substantial demands on families through the coordination of therapies, healthcare appointments, and educational support. Previous studies have shown that caregiver stress and QoL are strongly influenced by the availability of social support and mental health resources (Hayes and Watson 2013; Robinson and Weiss 2020; Dardas and Ahmad 2015). Taken together with the broader literature, our findings support the importance of considering family‐centered approaches when planning autism services. In contrast, South America shows the lowest mean scores for individuals with ASD but the highest perceived burden among caregivers, possibly indicating greater subjective overload in the face of structural limitations. These findings highlight the importance of considering local contexts in the planning of public policies and interventions aimed at both individuals with ASD and their caregivers.

In light of these findings, future research should prioritize longitudinal studies that track QoL and caregiver burden over time, particularly through major life transitions such as school entry, adolescence, and transition to adulthood. Such research could inform the development of scalable, sustainable support systems that address the evolving nature of ASD and caregiving demands. Furthermore, greater emphasis should be placed on cross‐cultural research to identify how systemic factors—such as healthcare infrastructure, disability policy, and cultural attitudes—shape caregiving experiences and access to support.

Finally, these findings underscore the need for further research using experimental and mixed‐methods designs with broader participant inclusion—particularly studies that directly involve individuals with ASD themselves. Such approaches are essential to deepen understanding of the factors that affect QoL and to develop more targeted, equitable, and sustainable interventions.

Despite the strengths of this review, including comprehensive database coverage and rigorous inclusion criteria, some limitations must be acknowledged. First, heterogeneity was high across most analyses, which limits the precision of the pooled estimates. Therefore, the findings should be interpreted as broad indicators of QoL patterns rather than precise population estimates. The observed heterogeneity likely reflects differences in study populations, assessment instruments, respondent types, and sociocultural contexts. Additionally, the exclusion of studies not published in English, Spanish, Portuguese, or French may have led to a geographic bias, underrepresenting data from some regions. Moreover, only a limited number of studies have adopted this specific approach or included comparisons with control groups, and there are no studies with globally representative samples or even comprehensive analyses at the continental level. Another important limitation relates to the use of different QoL assessment instruments. Although most scores could be converted to a common 0–100 metric, allowing clinically interpretable pooled estimates, differences in instrument structure, domains assessed, and respondent types may have contributed to the observed heterogeneity and should be considered when interpreting the findings.

## 5 Conclusion

This systematic review and meta‐analysis highlights the significant and persistent reduction in QoL among individuals with ASD and their caregivers, particularly in emotional and social domains. These impairments are not temporary but evolve over time, shaped by psychological, developmental, and contextual factors. Caregivers, in particular, experience high levels of stress, often accompanied by anxiety and depression, underscoring the urgent need for targeted support. While some psychosocial and educational interventions show promise, their effectiveness varies, suggesting the need for personalized, culturally sensitive, and multidimensional strategies. Examples include structured transition programs from pediatric to adult services, community‐based vocational training and supported employment initiatives, caregiver support groups, and multidisciplinary community networks that facilitate social participation and independent living. Interventions must also consider life transitions, such as adolescence to adulthood, which are often marked by service gaps and increased uncertainty. Moreover, structural factors, including national development and access to services, significantly influence QoL outcomes, particularly in low‐resource settings. The findings emphasize the importance of integrated efforts at both the clinical and policy levels to support these populations. Future research should adopt longitudinal and intervention‐focused designs, ensuring the inclusion of individuals with ASD to better tailor strategies to their needs. Ultimately, enhancing QoL for individuals with ASD and their caregivers is a public health priority with wide‐reaching implications for family well‐being and social inclusion.

## Author Contributions

K.C., C.K.D., E.S., L.H., and J.S.V. conceived the study and led the conceptualization and methodology. K.C. and C.K.D. conducted the formal analysis and data synthesis. Investigation, including data screening and extraction, was carried out by K.C, E.S., L.H., and C.K.D. The original draft of the manuscript was written by K.C., C.K.D., E.S., and L.H., with critical review and editing by all authors. All authors had full access to all the data in the study and verified the underlying data. All authors had full access to the data, reviewed the manuscript for important intellectual content, and had final responsibility for the decision to submit for publication.

## Funding

This study was funded by the Brazilian National Council for Scientific and Technological Development (CNPq), in partnership with the Department of Science and Technology of the Brazilian Ministry of Health (DECIT/MoH), under Grant No. 444574/2023‐9. This study was also financed in part by the Coordination for the Improvement of Higher Education Personnel (CAPES), Finance Code 001, which supported graduate scholarships for E.S., J.L.B., and L.V.H. J.S.V. is a research fellow of the Brazilian National Council for Scientific and Technological Development (CNPq).

## Conflicts of Interest

The authors declare no conflicts of interest.

## Supporting information


**Appendix S1:** Full search strategy.
**Table S1:** PECO Criteria for developing the search strategy.
**Table S2:** Search strategy used for databases and preprint repositories.
**Appendix S2:** Risk of bias assessments.
**S2.1:** Quality assessment in longitudinal studies (Newcastle‐Ottawa Scale—Cohort Studies).
**S2.2:** Quality assessment in cross‐sectional studies (Newcastle‐Ottawa Scale—Cross‐Sectional Studies).
**S2.3:** Quality assessment in randomized controlled trials (ROB‐2 Tool).
**S2.4:** Quality assessment in non‐randomized studies (ROBINS‐I Tool).
**Appendix S3:** List of excluded studies and reasons for exclusion in full‐text screening.
**Appendix S4:** Meta analysis—Forest Plot.
**Appendix S5:** Geographic distribution of mean quality of life scores for caregivers (a) and individuals with ASD (b), by continent.
**Appendix S6:** Complete list of references included in the review.

## Data Availability

The data that supports the findings of this study are available in the Supporting Information of this article.
