# Supplementary material for: Quality of Life in Autism: A Systematic Review and Meta‐Analysis of Patients and Caregivers
Source: Autism Res. 2026 Aug 24;19(10):e70348. doi: 10.1002/aur.70348 (PMC13674377; doi:10.1002/aur.70348)
Supplement: Supplementary file 1 [file AUR-19-0-s001.docx]

**Review Article**

Quality of Life in Autism: A Systematic Review and Meta‐Analysis of Patients and Caregivers

Castro et al., 2026

**Supplementary Appendix**

**Appendix S1. Full Search Strategy**

| **Table S1. PECO Criteria for developing the search strategy.** | |
| --- | --- |
| **Parameter** | **Description** |
| Population | Children/adolescents or caregivers (parents, relatives) |
| Exposure | Autism spectrum disorder |
| Comparison | Children and adolescents without ASD and/or their caregivers, when available |
| Outcome | Quality of life |

| **Table S2. Search strategy used for databases and preprint repositories.** | |
| --- | --- |
| **Database/**  **Preprint repository** | **Search strategy** |
| PubMed | (("Child"[Mesh] OR (Child) OR (Children) OR "Child, Preschool"[Mesh] OR (Preschool Child) OR (Children, Preschool) OR (Preschool Children) OR "Infant"[Mesh] OR (Infant) OR (Infants) OR "Adolescent"[Mesh] OR (Adolescent) OR (Adolescents) OR (Adolescence) OR (Teens) OR (Teen) OR (Teenagers) OR (Teenager) OR (Youth) OR (Youths) OR (Adolescents, Female) OR (Adolescent, Female) OR (Female Adolescent) OR (Female Adolescents) OR (Adolescents, Male) OR (Adolescent, Male) OR (Male Adolescent) OR (Male Adolescents) OR "Caregivers"[Mesh] OR (Caregivers) OR (Caregiver) OR (Carers) OR (Carer) OR (Care Givers) OR (Care Giver) OR (Family Caregivers) OR (Caregiver, Family) OR (Caregivers, Family) OR (Family Caregiver) OR "Parents"[Mesh] OR (Parents) OR (Parent) OR (Step-Parents) OR (Step Parents) OR (Step-Parent) OR (Stepparent) OR (Stepparents) OR (Parental Age) OR (Age, Parental) OR (Ages, Parental) OR (Parental Ages) OR "Fathers"[Mesh] OR (Fathers) OR (Father) OR "Mothers"[Mesh] OR (Mothers) OR (Mother) OR "Family"[Mesh] OR (Family) OR (Families) OR (Family Members) OR (Family Member) OR (Relatives) OR (Filiation) OR (Kinship Networks) OR (Kinship Network) OR (Network, Kinship) OR (Networks, Kinship) OR "Single Parent"[Mesh] OR (Single Parent) OR (Parent, Single) OR (Parents, Single) OR (Single Parents) OR (Single Stepparent) OR (Single Stepparents) OR (Stepparent, Single) OR (Stepparents, Single) OR (Single Step-Parent) OR (Single Step Parent) OR (Single Step-Parents) OR (Step-Parent, Single) OR (Step-Parents, Single)) AND ("Autistic Disorder"[Mesh] OR (Autistic Disorder) OR (Disorder, Autistic) OR (Disorders, Autistic) OR (Kanner's Syndrome) OR (Kanner Syndrome) OR (Kanners Syndrome) OR (Autism, Infantile) OR (Infantile Autism) OR (Autism) OR (Autism, Early Infantile) OR (Early Infantile Autism) OR (Infantile Autism, Early) OR "Autism Spectrum Disorder"[Mesh] OR (Autism Spectrum Disorder) OR (Autism Spectrum Disorders) OR (Autistic Spectrum Disorder) OR (Autistic Spectrum Disorders) OR (Disorder, Autistic Spectrum))) AND ("Quality of Life"[Mesh] OR (Quality of Life) OR (Life Quality) OR (Health-Related Quality of Life) OR (Health Related Quality of Life) OR (HRQOL) OR "Psychological Well-Being"[Mesh] OR (Psychological Well-Being) OR (Psychological Well Being) OR (Well-Being, Psychological) OR (Psychological Wellness) OR (Wellness, Psychological) OR (Psychological Ill-Being) OR (Ill-Being, Psychological) OR (Psychological Ill Being) OR "Sickness Impact Profile"[Mesh] OR (Sickness Impact Profile) OR (Impact Profile, Sickness) OR (Impact Profiles, Sickness) OR (Profile, Sickness Impact) OR (Profiles, Sickness Impact) OR (Sickness Impact Profiles) OR (Indicators of Quality of Life)) |
| SciELO | (Disorder, Autistic) OR (Disorders, Autistic) OR (Kanner's Syndrome) OR (Kanner Syndrome) OR (Kanners Syndrome) OR (Autism, Infantile) OR (Infantile Autism) OR (Autism) OR (Autism, Early Infantile) OR (Early Infantile Autism) OR (Infantile Autism, Early) OR (Autism Spectrum Disorder) OR (Autism Spectrum Disorders) OR (Autistic Spectrum Disorder) OR (Autistic Spectrum Disorders) OR (Disorder, Autistic Spectrum) |
| Scopus | TITLE-ABS-KEY((Children) OR (Preschool Child) OR (Children, Preschool) OR (Preschool Children) OR (Infants) OR (Adolescents) OR (Adolescence) OR (Teens) OR (Teen) OR (Teenagers) OR (Teenager) OR (Youth) OR (Youths) OR (Adolescents, Female) OR (Adolescent, Female) OR (Female Adolescent) OR (Female Adolescents) OR (Adolescents, Male) OR (Adolescent, Male) OR (Caregiver) OR (Carers) OR (Carer) OR (Care Givers) OR (Care Giver) OR (Family Caregivers) OR (Caregiver, Family) OR (Caregivers, Family) OR (Family Caregiver) OR (Parents) OR (Parent) OR (Step-Parents) OR (Step Parents) OR (Step-Parent) OR (Stepparent) OR (Stepparents) OR (Parental Age) OR (Age, Parental) OR (Ages, Parental) OR (Parental Ages) OR (Mother) OR (Father) OR (Families) OR (Family Members) OR (Family Member) OR (Relatives) OR (Filiation) OR (Kinship Networks) OR (Kinship Network) OR (Network, Kinship) OR (Networks, Kinship) OR (Parent, Single) OR (Parents, Single) OR (Single Parents) OR (Single Stepparent) OR (Single Stepparents) OR (Stepparent, Single) OR (Stepparents, Single) OR (Single Step-Parent) OR (Single Step Parent) OR (Single Step-Parents) OR (Step-Parent, Single) OR (Step-Parents, Single)) AND TITLE-ABS-KEY((Disorder, Autistic) OR (Disorders, Autistic) OR (Kanner's Syndrome) OR (Kanner Syndrome) OR (Kanners Syndrome) OR (Autism, Infantile) OR (Infantile Autism) OR (Autism) OR (Autism, Early Infantile) OR (Early Infantile Autism) OR (Infantile Autism, Early) OR (Autism Spectrum Disorders) OR (Autistic Spectrum Disorder) OR (Autistic Spectrum Disorders) OR (Disorder, Autistic Spectrum)) AND TITLE-ABS-KEY((Life Quality) OR (Health-Related Quality of Life) OR (Health Related Quality of Life) OR (HRQOL) OR (Psychological Well Being) OR (Well-Being, Psychological) OR (Psychological Wellness) OR (Wellness, Psychological) OR (Psychological Ill-Being) OR (Ill-Being, Psychological) OR (Psychological Ill Being) OR (Indicators of Quality of Life) OR (Impact Profile, Sickness) OR (Impact Profiles, Sickness) OR (Profile, Sickness Impact) OR (Profiles, Sickness Impact) OR (Sickness Impact Profiles)) |
| Embase | ('child'/syn OR 'infant'/syn OR 'adolescent'/syn OR 'adolescence'/syn OR 'caregiver'/syn OR 'formal caregiver'/syn OR 'informal caregiver'/syn OR 'parent'/syn OR 'adolescent parent'/syn OR 'father'/syn OR 'mother'/syn OR 'stepparent'/syn OR 'stepfather'/syn OR 'stepmother'/syn OR 'family'/syn OR 'kinship networks'/syn OR 'relative'/syn OR 'single parent'/syn OR 'separated parent'/syn OR 'parenthood'/syn) AND ('autism'/syn OR 'autism spectrum disorders'/syn) AND ('quality of life'/syn OR 'quality of life'/exp OR 'health related quality of life' OR 'HRQL' OR 'life quality' OR 'quality of life') |
| PsycINFO | (Kanner's Syndrome) OR (Kanner Syndrome) OR (Kanners Syndrome) OR (Infantile Autism) OR (Autism) OR (Early Infantile Autism) OR (Autism Spectrum Disorders) OR (Autistic Spectrum Disorder) OR (Autistic Spectrum Disorders) |
| BioRXiv | "autism" AND "quality of life" AND ("parents" OR "caregivers" OR "child" OR "children" OR "adolescents" OR "teenagers") |
| MedRXiv |  |
| OSF Preprints | "autism" AND "quality of life" |
| Preprints.org |  |
| PsyArXiv |  |

**Appendix S2. Risk of Bias Assessments**

| **S2.1 Quality Assessment in Longitudinal Studies (Newcastle-Ottawa Scale - Cohort Studies)** | | | | |
| --- | --- | --- | --- | --- |
| **Study design** | **Quality Assessment Tool** | **Study** | **Risk of Bias** |  |
| Longitudinal | NOS - Cohort Studies | Clark & Dissanayake, 2024 | 5 |  |
| Longitudinal | NOS - Cohort Studies | Schneider et al., 2022 | 4 |  |
| Longitudinal | NOS - Cohort Studies | Adams et al., 2019 | 3 |  |
| Longitudinal | NOS - Cohort Studies | Bent et al., 2020 | 4 |  |
| Longitudinal | NOS - Cohort Studies | Kuhlthau et al., 2017 | 5 |  |
| Longitudinal | NOS - Cohort Studies | Dai et al., 2024 | 4 |  |

| **S2.2 Quality Assessment in Cross-Sectional Studies (Newcastle-Ottawa Scale - Cross-Sectional Studies)** | | | | |
| --- | --- | --- | --- | --- |
| **Study design** | **Quality Assessment Tool** | **Study** | **Risk of Bias** |  |
| Cross-sectional | NOS - Cross-Sectional Studies | Kousha et al., 2019 | 5 |  |
| Cross-sectional | NOS - Cross-Sectional Studies | Tekinarslan, 2013 | 6 |  |
| Cross-sectional | NOS - Cross-Sectional Studies | Bulkley et al., 2023 | 8 |  |
| Cross-sectional | NOS - Cross-Sectional Studies | Due et al., 2017 | 2 |  |
| Cross-sectional | NOS - Cross-Sectional Studies | Amirian et al., 2017 | 5 |  |
| Cross-sectional | NOS - Cross-Sectional Studies | Derguy et al., 2018 | 5 |  |
| Cross-sectional | NOS - Cross-Sectional Studies | Howe et al., 2023 | 4 |  |
| Cross-sectional | NOS - Cross-Sectional Studies | Kousha et al., 2015 | 6 |  |
| Cross-sectional | NOS - Cross-Sectional Studies | Willet et al., 2018 | 2 |  |
| Cross-sectional | NOS - Cross-Sectional Studies | Zeng et al., 2020 | 5 |  |
| Cross-sectional | NOS - Cross-Sectional Studies | Khanna et al., 2011 | 4 |  |
| Cross-sectional | NOS - Cross-Sectional Studies | ABD-Alnabi & Sachet, 2022 | 1 |  |
| Cross-sectional | NOS - Cross-Sectional Studies | Martin et al., 2021 | 5 |  |
| Cross-sectional | NOS - Cross-Sectional Studies | Menezes & Mazurek, 2021 | 6 |  |
| Cross-sectional | NOS - Cross-Sectional Studies | Herpers et al., 2015 | 6 |  |
| Cross-sectional | NOS - Cross-Sectional Studies | Sari et al., 2022 | 2 |  |
| Cross-sectional | NOS - Cross-Sectional Studies | Hedgecock, et al., 2018 | 6 |  |
| Cross-sectional | NOS - Cross-Sectional Studies | Sikora et al., 2012 | 7 |  |
| Cross-sectional | NOS - Cross-Sectional Studies | Leader et al., 2022 | 4 |  |
| Cross-sectional | NOS - Cross-Sectional Studies | May et al., 2020 | 7 |  |
| Cross-sectional | NOS - Cross-Sectional Studies | Jones et al., 2017 | 5 |  |
| Cross-sectional | NOS - Cross-Sectional Studies | Randall et al., 2015 | 7 |  |
| Cross-sectional | NOS - Cross-Sectional Studies | Albadry et al., 2023 | 2 |  |
| Cross-sectional | NOS - Cross-Sectional Studies | Lopez-Espejo et al., 2021 | 6 |  |
| Cross-sectional | NOS - Cross-Sectional Studies | Patel et al., 2022 | 4 |  |
| Cross-sectional | NOS - Cross-Sectional Studies | Naheed et al., 2019 | 6 |  |
| Cross-sectional | NOS - Cross-Sectional Studies | Hand et al., 2021 | 6 |  |
| Cross-sectional | NOS - Cross-Sectional Studies | Lopes-Espejo et al., 2023 | 5 |  |
| Cross-sectional | NOS - Cross-Sectional Studies | ten Hoopen et al., 2021 | 7 |  |
| Cross-sectional | NOS - Cross-Sectional Studies | ten Hoopen et al., 2020 | 7 |  |
| Cross-sectional | NOS - Cross-Sectional Studies | Jahan et al., 2015 | 4 |  |
| Cross-sectional | NOS - Cross-Sectional Studies | Mazon et al., 2019 | 9 |  |
| Cross-sectional | NOS - Cross-Sectional Studies | Leader et al., 2021 | 3 |  |
| Cross-sectional | NOS - Cross-Sectional Studies | Leader et al., 2021 | 3 |  |
| Cross-sectional | NOS - Cross-Sectional Studies | Gabra & Hashem, 2021 | 4 |  |
| Cross-sectional | NOS - Cross-Sectional Studies | Kheir et al., 2012 | 4 |  |
| Cross-sectional | NOS - Cross-Sectional Studies | Vaz et al., 2021 | 7 |  |
| Cross-sectional | NOS - Cross-Sectional Studies | Dardas & Ahmad, 2013 | 5 |  |
| Cross-sectional | NOS - Cross-Sectional Studies | Tung et al., 2014 | 4 |  |
| Cross-sectional | NOS - Cross-Sectional Studies | Lei & Kantor, 2023 | 4 |  |
| Cross-sectional | NOS - Cross-Sectional Studies | Kilincaslan et al., 2019 | 6 |  |
| Cross-sectional | NOS - Cross-Sectional Studies | Fávero-Nunes & Santos, 2010 | 4 |  |
| Cross-sectional | NOS - Cross-Sectional Studies | Öz et al., 2020 | 2 |  |
| Cross-sectional | NOS - Cross-Sectional Studies | Flor et al., 2017 | 8 |  |
| Cross-sectional | NOS - Cross-Sectional Studies | Green et al., 2021 | 5 |  |
| Cross-sectional | NOS - Cross-Sectional Studies | Öztürk et al., 2016 | 5 |  |
| Cross-sectional | NOS - Cross-Sectional Studies | Alenazi et al., 2020 | 2 |  |
| Cross-sectional | NOS - Cross-Sectional Studies | Waheed et al., 2020 | 3 |  |
| Cross-sectional | NOS - Cross-Sectional Studies | Russell et al., 2023 | 6 |  |
| Cross-sectional | NOS - Cross-Sectional Studies | Masuri et al., 2023 | 1 |  |
| Cross-sectional | NOS - Cross-Sectional Studies | Papadopoulos et al., 2023 | 4 |  |
| Cross-sectional | NOS - Cross-Sectional Studies | Fourcade et al., 2015 | 1 |  |
| Cross-sectional | NOS - Cross-Sectional Studies | Dardas et al., 2015 | 4 |  |
| Cross-sectional | NOS - Cross-Sectional Studies | McStay et al., 2014 | 6 |  |
| Cross-sectional | NOS - Cross-Sectional Studies | McStay et al., 2014 | 6 |  |
| Cross-sectional | NOS - Cross-Sectional Studies | Domellöf et al., 2014 | 4 |  |
| Cross-sectional | NOS - Cross-Sectional Studies | Lee et al., 2018 | 4 |  |
| Cross-sectional | NOS - Cross-Sectional Studies | Güneş & Ekinci, 2019 | 5 |  |
| Cross-sectional | NOS - Cross-Sectional Studies | Wang et al., 2021 | 5 |  |
| Cross-sectional | NOS - Cross-Sectional Studies | Losada-Puente et al., 2022 | 3 |  |
| Cross-sectional | NOS - Cross-Sectional Studies | Feng et al., 2022 | 6 |  |
| Cross-sectional | NOS - Cross-Sectional Studies | Beheshti et al., 2022 | 3 |  |
| Cross-sectional | NOS - Cross-Sectional Studies | Ozboke et al., 2022 | 5 |  |
| Cross-sectional | NOS - Cross-Sectional Studies | Stiller et al., 2024 | 3 |  |
| Cross-sectional | NOS - Cross-Sectional Studies | Anixt et al., 2020 | 6 |  |
| Cross-sectional | NOS - Cross-Sectional Studies | Özgür et al., 2018 | 4 |  |
| Cross-sectional | NOS - Cross-Sectional Studies | Kuhlthau et al., 2013 | 5 |  |
| Cross-sectional | NOS - Cross-Sectional Studies | Kose et al., 2013 | 7 |  |
| Cross-sectional | NOS - Cross-Sectional Studies | Rizk et al., 2012 | 3 |  |
| Cross-sectional | NOS - Cross-Sectional Studies | Kuhlthau et al., 2010 | 5 |  |
| Cross-sectional | NOS - Cross-Sectional Studies | Chaabène et al., 2018 | 4 |  |
| Cross-sectional | NOS - Cross-Sectional Studies | Pavlova et al., 2021 | 3 |  |
| Cross-sectional | NOS - Cross-Sectional Studies | Ye et al., 2024 | 5 |  |
| Cross-sectional | NOS - Cross-Sectional Studies | Mannion & Leader, 2023 | 5 |  |
| Cross-sectional | NOS - Cross-Sectional Studies | Lanyi et al., 2022 | 4 |  |
| Cross-sectional | NOS - Cross-Sectional Studies | Siah & Tan, 2016 | 4 |  |
| Cross-sectional | NOS - Cross-Sectional Studies | Cardelle-Perez et al., 2024 | 3 |  |
| Cross-sectional | NOS - Cross-Sectional Studies | Logrieco et al., 2022 | 5 |  |
| Cross-sectional | NOS - Cross-Sectional Studies | Saigh & Bagadood, 2022 | 3 |  |
| Cross-sectional | NOS - Cross-Sectional Studies | Siah & Tan, 2015 | 4 |  |
| Cross-sectional | NOS - Cross-Sectional Studies | Ying et al., 2018 | 6 |  |
| Cross-sectional | NOS - Cross-Sectional Studies | Lei & Kantor, 2022 | 4 |  |
| Cross-sectional | NOS - Cross-Sectional Studies | Schlebusch et al., 2016 | 4 |  |
| Cross-sectional | NOS - Cross-Sectional Studies | Selvakumar & Panicker, 2020 | 5 |  |
| Cross-sectional | NOS - Cross-Sectional Studies | McStay et al., 2014 | 7 |  |
| Cross-sectional | NOS - Cross-Sectional Studies | Parsons et al., 2020 | 5 |  |
| Cross-sectional | NOS - Cross-Sectional Studies | Lee et al., 2012 | 5 |  |
| Cross-sectional | NOS - Cross-Sectional Studies | Werner & Shulman, 2013 | 4 |  |
| Cross-sectional | NOS - Cross-Sectional Studies | Russell et al., 2017 | 5 |  |
| Cross-sectional | NOS - Cross-Sectional Studies | Vahedparast et al., 2022 | 3 |  |
| Cross-sectional | NOS - Cross-Sectional Studies | Eskow et al., 2018 | 6 |  |
| Cross-sectional | NOS - Cross-Sectional Studies | Gabra et al., 2021 | 6 |  |
| Cross-sectional | NOS - Cross-Sectional Studies | Adams et al., 2020 | 2 |  |
| Cross-sectional | NOS - Cross-Sectional Studies | Herrera et al., 2022 | 3 |  |
| Cross-sectional | NOS - Cross-Sectional Studies | Delahaye et al., 2014 | 5 |  |
| Cross-sectional | NOS - Cross-Sectional Studies | Süzen et al., 2022 | 2 |  |
| Cross-sectional | NOS - Cross-Sectional Studies | Kinter et al., 2024 | 6 |  |
| Cross-sectional | NOS - Cross-Sectional Studies | Tait et al., 2016 | 3 |  |
| Cross-sectional | NOS - Cross-Sectional Studies | Clark et al., 2015 | 4 |  |
| Cross-sectional | NOS - Cross-Sectional Studies | Garrido et al., 2020 | 5 |  |
| Cross-sectional | NOS - Cross-Sectional Studies | Elias et al., 2006 | 5 |  |
| Cross-sectional | NOS - Cross-Sectional Studies | Lal D V et al., 2020 | 7 |  |
| Cross-sectional | NOS - Cross-Sectional Studies | Kuru et al., 2018 | 4 |  |
| Cross-sectional | NOS - Cross-Sectional Studies | Sorrenti et al., 2021 | 3 |  |
| Cross-sectional | NOS - Cross-Sectional Studies | Mahjoob et al., 2024 | 7 |  |
| Cross-sectional | NOS - Cross-Sectional Studies | Ji et al., 2014 | 7 |  |
| Cross-sectional | NOS - Cross-Sectional Studies | Dardas & Ahmad, 2014 | 7 |  |
| Cross-sectional | NOS - Cross-Sectional Studies | Chuang et al., 2014 | 5 |  |
| Cross-sectional | NOS - Cross-Sectional Studies | Tilford et al., 2012 | 6 |  |
| Cross-sectional | NOS - Cross-Sectional Studies | Dardas & Ahmad, 2014 | 5 |  |
| Cross-sectional | NOS - Cross-Sectional Studies | Barbosa et al., 2009 | 4 |  |
| Cross-sectional | NOS - Cross-Sectional Studies | Al-Farsi et al., 2022 | 8 |  |
| Cross-sectional | NOS - Cross-Sectional Studies | Ismail et al., 2022 | 4 |  |
| Cross-sectional | NOS - Cross-Sectional Studies | Dardas; Ahmad, 2014 | 6 |  |
| Cross-sectional | NOS - Cross-Sectional Studies | Dardas; Ahmad, 2014 | 5 |  |
| Cross-sectional | NOS - Cross-Sectional Studies | Alhazmi et al., 2018 | 6 |  |
| Cross-sectional | NOS - Cross-Sectional Studies | Asahar et al., 2021 | 6 |  |
| Cross-sectional | NOS - Cross-Sectional Studies | ALBusaidi et al., 2022 | 4 |  |
| Cross-sectional | NOS - Cross-Sectional Studies | Soccorso et al., 2023 | 5 |  |
| Cross-sectional | NOS - Cross-Sectional Studies | Leonard et al., 2022 | 4 |  |
| Cross-sectional | NOS - Cross-Sectional Studies | Pecor et al., 2021 | 3 |  |
| Cross-sectional | NOS - Cross-Sectional Studies | Kuhlthau et al., 2014 | 6 |  |
| Cross-sectional | NOS - Cross-Sectional Studies | Perumal et al., 2014 | 5 |  |
| Cross-sectional | NOS - Cross-Sectional Studies | Mello et al., 2019 | 6 |  |
| Cross-sectional | NOS - Cross-Sectional Studies | Millau et al., 2019 | 4 |  |
| Cross-sectional | NOS - Cross-Sectional Studies | Eapen et al., 2023 | 6 |  |
| Cross-sectional | NOS - Cross-Sectional Studies | Durhan et al., 2024 | 2 |  |
| Cross-sectional | NOS - Cross-Sectional Studies | Raju et al., 2023 | 4 |  |
| Cross-sectional | NOS - Cross-Sectional Studies | Sheldrick et al., 2012 | 2 |  |
| Cross-sectional | NOS - Cross-Sectional Studies | Jain et al., 2019 | 4 |  |
| Cross-sectional | NOS - Cross-Sectional Studies | Kheir et al., 2012 | 7 |  |
| Cross-sectional | NOS - Cross-Sectional Studies | Renford et al., 2020 | 4 |  |
| Cross-sectional | NOS - Cross-Sectional Studies | Shu, 2009 | 3 |  |
| Cross-sectional | NOS - Cross-Sectional Studies | Egilson et al., 2016 | 6 |  |
| Cross-sectional | NOS - Cross-Sectional Studies | Ahmed et al., 2023 | 5 |  |
| Cross-sectional | NOS - Cross-Sectional Studies | Poirier et al., 2018 | 3 |  |
| Cross-sectional | NOS - Cross-Sectional Studies | McAuliffe et al., 2017 | 3 |  |
| Cross-sectional | NOS - Cross-Sectional Studies | Celik et al., 2021 | 6 |  |
| Cross-sectional | NOS - Cross-Sectional Studies | Oakley et al., 2020 | 7 |  |
| Cross-sectional | NOS - Cross-Sectional Studies | Williams et al., 2020 | 3 |  |
| Cross-sectional | NOS - Cross-Sectional Studies | De Castro et al., 2022 | 5 |  |
| Cross-sectional | NOS - Cross-Sectional Studies | Likhitweeranwong et al., 2020 | 7 |  |
| Cross-sectional | NOS - Cross-Sectional Studies | Coales et al., 2019 | 9 |  |
| Cross-sectional | NOS - Cross-Sectional Studies | Lara & Los Pinos, 2015 | 3 |  |
| Cross-sectional | NOS - Cross-Sectional Studies | Meral et al., 2013 | 5 |  |
| Cross-sectional | NOS - Cross-Sectional Studies | Eskow et al., 2011 | 4 |  |
| Cross-sectional | NOS - Cross-Sectional Studies | Ran et al., 2023 | 4 |  |
| Cross-sectional | NOS - Cross-Sectional Studies | Davy et al., 2024 | 6 |  |
| Cross-sectional | NOS - Cross-Sectional Studies | Eapen et al., 2022 | 4 |  |
| Cross-sectional | NOS - Cross-Sectional Studies | Wang et al., 2020 | 5 |  |
| Cross-sectional | NOS - Cross-Sectional Studies | Musetti et al., 2024 | 4 |  |
| Cross-sectional | NOS - Cross-Sectional Studies | Gardier & Iarocci, 2015 | 4 |  |
| Cross-sectional | NOS - Cross-Sectional Studies | Moretto et al., 2020 | 4 |  |
| Cross-sectional | NOS - Cross-Sectional Studies | Romaniuk et al., 2022 | 7 |  |
| Cross-sectional | NOS - Cross-Sectional Studies | Schlebusch et al., 2017 | 4 |  |
| Cross-sectional | NOS - Cross-Sectional Studies | Zeng et al., 2019 | 5 |  |
| Cross-sectional | NOS - Cross-Sectional Studies | Leader et al., 2021 | 5 |  |
| Cross-sectional | NOS - Cross-Sectional Studies | Tavernor et al., 2012 | 1 |  |
| Cross-sectional | NOS - Cross-Sectional Studies | Katsiana et al., 2020 | 5 |  |
| Cross-sectional | NOS - Cross-Sectional Studies | Salami & Alhalal., 2024 | 4 |  |
| Cross-sectional | NOS - Cross-Sectional Studies | Jamison & Shuttler, 2015 | 4 |  |
| Cross-sectional | NOS - Cross-Sectional Studies | AlHefdhi et al., 2024 | 4 |  |
| Cross-sectional | NOS - Cross-Sectional Studies | Forouzanfar et al., 2016 | 2 |  |
| Cross-sectional | NOS - Cross-Sectional Studies | Nuske et al., 2017 | 7 |  |
| Cross-sectional | NOS - Cross-Sectional Studies | Lee et al., 2009 | 5 |  |
| Cross-sectional | NOS - Cross-Sectional Studies | Schertz et al., 2015 | 5 |  |
| Cross-sectional | NOS - Cross-Sectional Studies | Alhuzimi, 2021 | 3 |  |
| Validation Study | NOS - Cross-Sectional Studies | Chezan et al., 2021 | 5 |  |
| Validation Study | NOS - Cross-Sectional Studies | Hoefman et al., 2015 | 3 |  |
| Validation Study | NOS - Cross-Sectional Studies | Eapen et al., 2014 | 6 |  |
| Validation Study | NOS - Cross-Sectional Studies | Lichtlé et al., 2023 | 4 |  |
| Validation Study | NOS - Cross-Sectional Studies | Chezan et al., 2023 | 3 |  |
| Validation Study | NOS - Cross-Sectional Studies | Khanna et al., 2012 | 4 |  |
| Validation Study | NOS - Cross-Sectional Studies | Pratesi et al., 2021 | 5 |  |
| Cross-sectional | NOS - Cross-Sectional Studies | Shipman et al., 2011 | 3 |  |
| Cross-sectional | NOS - Cross-Sectional Studies | Robeson et al., 2023 | 4 |  |
| Cross-sectional | NOS - Cross-Sectional Studies | Fayyz et al., 2023 | 3 |  |
| Cross-sectional | NOS - Cross-Sectional Studies | Lee et al., 2023 | 3 |  |
| Cross-sectional | NOS - Cross-Sectional Studies | Broquere et al., 2016 | 6 |  |
| Cross-sectional | NOS - Cross-Sectional Studies | Vries; Geurts, 2015 | 7 |  |
| Cross-sectional | NOS - Cross-Sectional Studies | Potvin et al., 2015 | 6 |  |
| Cross-sectional | NOS - Cross-Sectional Studies | Pisula et al., 2017 | 7 |  |
| Cross-sectional | NOS - Cross-Sectional Studies | Günal et al., 2019 | 5 |  |
| Cross-sectional | NOS - Cross-Sectional Studies | Taylor et al., 2021 | 6 |  |

| **S2.3 Quality Assessment in Randomized Controlled Trials (ROB-2 Tool)** | | | | |
| --- | --- | --- | --- | --- |
| **Study design** | **Quality Assessment Tool** | **Study** | **Risk of Bias** |  |
| Randomized controlled trial | ROB-2 | Roberts et al., 2011 | Some concerns |  |
| Randomized controlled trial | ROB-2 | Green et al., 2022 | Low |  |
| Randomized controlled trial | ROB-2 | Varni et al., 2012 | Some concerns |  |
| Randomized controlled trial | ROB-2 | Prasitwut et al., 2024 | Some concerns |  |
| Randomized controlled trial | ROB-2 | Zhao et al., 2021 | Some concerns |  |
| Randomized controlled trial | ROB-2 | Ji et al., 2014 | High |  |
| Randomized controlled trial | ROB-2 | Kim et al., 2022 | Low |  |
| Randomized controlled trial | ROB-2 | Vries et al., 2017 | Some concerns |  |
| Randomized controlled trial | ROB-2 | Toscano et al., 2017 | High |  |
| Randomized controlled trial | ROB-2 | Akhani et al., 2021 | Some concerns |  |
| Randomized controlled trial | ROB-2 | Palmer et al., 2024 | Low |  |

| **S2.4 Quality Assessment in Non-randomized studies (ROBINS-I Tool)** | | | | |
| --- | --- | --- | --- | --- |
| **Study design** | **Quality Assessment Tool** | **Study** | **Risk of Bias** |  |
| Non-randomized trial | ROBINS-I | van Steensel & Bögels, 2015 | Serious |  |
| Non-randomized trial | ROBINS-I | Roberts et al., 2019 | Moderate |  |
| Non-randomized trial | ROBINS-I | Cerit et al., 2022 | Serious |  |
| Non-randomized trial | ROBINS-I | Rayan & Ahmad, 2016 | Serious |  |
| Non-randomized trial | ROBINS-I | Gengoux et al., 2018 | Moderate |  |
| Non-randomized trial | ROBINS-I | Lanning et al., 2014 | Serious |  |
| Non-randomized trial | ROBINS-I | Naheed et al., 2022 | Moderate |  |
| Non-randomized trial | ROBINS-I | Cappe et al., 2021 | Moderate |  |
| Non-randomized trial | ROBINS-I | Chiang, 2014 | Moderate |  |
| Non-randomized trial | ROBINS-I | Drüsedeau et al, 2019 | Serious |  |
| Non-randomized trial | ROBINS-I | Al-Masa'deh et al., 2022 | Serious |  |
| Non-randomized trial | ROBINS-I | Sarol & Çimen, 2015 | Moderate |  |
| Non-randomized trial | ROBINS-I | Hatfield et al., 2017 | Serious |  |
| Non-randomized trial | ROBINS-I | Biscaldi-Schafer et al., 2016 | Moderate |  |

| **Appendix S3**. List of excluded studies and reasons for exclusion in full-text screening | |
| --- | --- |
| **Exclusion reason** | **References** |
| Out of scope (N= 121) | Abbeduto et al., 2004; Afsharnejad et al., 2020; Al-Jabri et al., 2022; Alasiri et al., 2024; Alnahdi et al., 2021; Arias et al., 2018; Arnold et al., 2019; Ayça et al., 2021; Azad et al., 2020; Baghdadli et al., 2014; Balcells-Balcells et al., 2020; Bangma et al., 2018; Barendregt et al., 2015; Barros et al., 2019; Benjak, 2011; Bent et al., 2020; Błeszyński, 2018; Bolbocea et al., 2022; Bolbocean et al., 2020; Bolbocean et al., 2022; Bolbocean et al., 2023; Borissov et al., 2022; Bourke-Taylor et al., 2010; Brandsma et al., 2024; Burgess & Turkstra, 2010; Cappe et al., 2011; Cappe et al., 2020; Cardoso et al., 2023; Chan et al., 2019; Cheng et al., 2022; Chezan et al., 2023; Cholewicki et al., 2019; Çoban et al., 2022; Costa et al., 2017; Cottenceau et al., 2012; Danhofer et al., 2020; DaWalt et al., 2019; de Azevedo et al., 2019; de Leeuw et al., 2024; Downs et al., 2019; Epstein et al., 2019; Eskow et al., 2015; Eskow et al., 2019; Fernández et al., 2021; Fong et al., 2020; Frazier et al., 2020; Frazier et al., 2022; García et al., 2021; García et al., 2022; García et al., 2022; Gardiner et al., 2019; Gómez et al., 2020; Hartley et al., 2012; Hatta et al., 2019; Hsiao et al., 2017; Hsiao et al., 2018; Hsiao, 2016; Hwang et al., 2015; Isa et al., 2021; Jose et al., 2017; Kaba et al., 2023; Kamp-Becker et al., 2010; Knüppel et al., 2018; Knüppel et al., 2018; Laufer & Isman, 2024; Leadbitter et al., 2018; Lu et al., 2018; Mahfouda et al., 2019; Marsack-Topolewski, 2021; McClean; Grey, 2012; McConachie et al., 2018; Mello et al., 2023; Meral et al., 2021; Meral & Fidan, 2015; Mills et al., 2021; Misquiatti et al., 2015; Mumbardó-Adam et al., 2023; Muñoz et al., 2023; Nelson et al., 2023; Niinomi et al., 2016; Özgur et al., 2017; Ozturk et al., 2023; Papadopoulou et al., 2015; Payakachat et al., 2014; Pejovic-Milovancevic et al., 2018; Perry et al., 2024; Perry & Isaacs, 2015; Piovesan et al., 2015; Pozo et al., 2014; Puthiyapurayil et al., 2022; Qiao et al., 2020; Raghunandan et al., 2023; Redd et al., 2016; Reed & Osborne, 2018; Rivard et al., 2017; Rivard et al., 2023; Schmidt et al., 2017; Schoder et al., 2019; Shamsudin et al., 2019; Singh et al., 2020; Staunton et al., 2020; Stephens et al., 2021; Stokes et al., 2017; Suárez et al., 2019; Suen et al., 2024; Tait & Hussain, 2017; Telman et al., 2017; Thomas et al., 2018; Tien et al., 2022; van Steensel et al., 2012; van Tongerloo et al., 2014; Vernhet et al., 2022; Viecili et al., 2015; Volgyesi-Molnar et al., 2024; Wang et al., 2006; Weyland et al., 2022; Whitehouse et al., 2021; Yildiz et al., 2016; Yildiz et al., 2023; Yousefi et al., 2015; Zarbo et al., 2016 |
| Abstract, chapter, review, and short communication (N=50) | Alhazmi et al., 2017; Amr et al., 2012; Aral et al., 2015; Barbayannis et al., 2021; Bernardo & Santos, 2013; Brugnaro et al., 2022; Byars et al., 2013; Calonge-Torres et al., 2017; Choi, 2021; Church & Dyson, 2016; Deshpande et al., 2019; Downs et al., 2019; Downs et al., 2022; Dunbar-Masterson et al., 2014; Epstein et al., 2016; Epstein et al., 2018; Epstein et al., 2020; Erermis et al., 2012; Furusho et al., 2006; Gomez et al., 2016; Haj Ebrahimi et al., 2015; Isaacs & Perry, 2012; Katz et al., 2013; Kirkpatrick & Leader, 2018; Kocas et al., 2014; Kousha & Attar, 2012; Kumar, 2019; Lai et al., 2016; Lope et al., 2013; Lopez-Espejo et al., 2021; Malow et al., 2012; Mohammadi et al., 2012; Moussa et al., 2019; Ozal et al., 2020; Ozturk et al., 2012; Patel et al., 2016; Patil & Jaiswal, 2011; Radu et al., 2020; Rivard et al., 2019; Russell et al., 2015; Schoemaker & Houwen, 2021; Shah et al., 2023; Sipos et al., 2012; Srivastava et al., 2019; Suda, 2016; Vassiliki et al., 2013; Voloshyn et al., 2020; Williams et al., 2010; Wisessathorn et al., 2013; Zamani et al., 2022 |
| Non-validated or qualitative tools (N=44) | Almasoud & Alqahtani, 2023; Amirova et al., 2022; Aun et al., 2022; Baghdadli et al., 2014; Bar et al., 2023; Begeer et al., 2017; Brillet et al., 2023; Cappe et al., 2012; Cappe et al., 2018; Cappe, 2012; Chu, et al., 2020; Cuesta-Gómez et al., 2016; Cuesta-Gómez et al., 2022; Epstein et al., 2019; Fante et al., 2024; Fewster et al., 2020; Folostina et al., 2023; Frazier et al., 2017; Gagat-Matula, 2021; García-Gómez et al., 2014; Garrido et al., 2021; Gómez et al., 2018; Holmes et al., 2020; Hudock et al., 2024; Ikeda et al., 2016; Kasem et al., 2024; Lee et al., 2008; Li et al., 2022; Ljubičić et al., 2022; Mahdavi et al., 2023; Mai & Chaimongkol, 2022; McLaughlin et al., 2020; Minolin et al., 2019; Minolin et al., 2022; Nohelty et al., 2023; Ong & Goh, 2016; Pyszkowska & Górnik-Durose, 2023; Pyszkowska & Wrona, 2021; Rattaz et al., 2015; Rodríguez et al., 2020; Smith et al., 2021; Turda, 2023; Xu et al., 2019; Zablotsky et al., 2013 |
| Full text unavailable or language barrier (N= 11) | Abouzeid et al., 2019; Blanchon & Allouard, 1998; Chen et al., 2024; Derguy et al., 2018; Kamp-Becker et al., 2011; Kuznetsova et al., 2022; Montalbano & Rocella, 2009; Özyurt, et al., 2017; Taghvaei et al., 2019; Whitney et al., 2012; Yurteri & Akdemir, 2019 |
| Absent data (N= 4) | Garrido et al., 2015; Kiani & Nami, 2017; Maras et al., 2018; Windarwati et al., 2024 |

**Appendix S4. Meta analysis - Forest Plot**

a) Emotional domain – Caregivers

b) Emotional domain – ASD

c) Emotional domain – ASD per age group

d) Social domain – caregivers

e) Social domain – ASD

f) Social domain – ASD per age group

g) Physical domain – ASD

h) Physical domain – Caregivers

**Appendix S5. Geographic distribution of mean quality of life scores for caregivers (a) and individuals with ASD (b), by continent**

**Appendix S6. Complete list of references included in the review**

1.	ABD-Alnabi AL, Sachet KR. Assessment of Quality of Life among Parents with Autistic Child. Pakistan Journal of Medical & Health Sciences. 2022 Mar;16(3).

2.	Adams D, Clark M, Keen D. Using self-report to explore the relationship between anxiety and quality of life in children on the autism spectrum. Autism Res. 2019 Oct;12(10):1505–15.

3.	Adams D, Clark M, Simpson K. The Relationship Between Child Anxiety and the Quality of Life of Children, and Parents of Children, on the Autism Spectrum. J Autism Dev Disord. 2020 May;50(5):1756–69.

4.	Ahmed EA, Alkhaldi SM, Alduraidi H, Albsoul RA, Alhamdan MZ. Quality of Life of Mothers and Fathers of Children with Autism Spectrum Disorder in Jordan. Clin Pract Epidemiol Ment Health. 2023;19:e174501792304180.

5.	Akhani A, Dehghani M, Gharraee B, Hakim Shooshtari M. Parent training intervention for autism symptoms, functional emotional development, and parental stress in children with autism disorder: A randomized clinical trial. Asian J Psychiatr. 2021 Aug;62:102735.

6.	Al-Farsi OA, Al-Farsi YM, Al-Sharbati MM, Al-Adawi S, Cucchi A, Essa MM, et al. Quality of Life among Caregivers of children with Autism Spectrum Disorders, Intellectual Disability, and Typical Development. Applied Research Quality Life. 2022 Feb 1;17(1):129–45.

7.	Al-Masa’deh MM, Younis NA, Al-Zyoud NS, Homidi MA, Rahahleh ZJ. The Effectiveness of Counseling Programs in Enhancing Quality of Life for Families of Children with Autism Spectrum Disorder in Jordan. Humanities and Social Sciences Letters. 2022 Mar 25;10(1):75–87.

8.	Albadry ES, Abd-Elmonem AM, Othman AA, Abbass ME. Autistic features in relation to motor function and quality of life in children with autism spectrum disorder. Phys Act Rev. 2023;11(1):123–30.

9.	ALBusaidi AMS, ALMasroori SAS, Awladthani BMS, ALKhaldi SA, Al Alawi N, Al Salmani AA. Quality of life and coping strategies used by parents of children with autism spectrum disorder in Oman. Autism Dev Lang Impair. 2022;7:23969415221142262.

10.	Alenazi DS, Hammad SM, Mohamed AE. Effect of autism on parental quality of life in Arar city, Saudi Arabia. J Family Community Med. 2020;27(1):15–22.

11.	Alhazmi A, Petersen R, Donald KA. Quality of life among parents of South African children with autism spectrum disorder. Acta Neuropsychiatr. 2018 Aug;30(4):226–31.

12.	AlHefdhi HA, Al Zomia AS, Alshehri NM, Alaskari AA, Hussain AA, Lahiq LA, et al. Examining the Quality of Life and Discrimination Impact on Parents of Children With Autism Spectrum Disorder in Aseer Region, Saudi Arabia: A WHO-QOL Survey. Cureus. 2024 Feb;16(2):e53616.

13.	Alhuzimi T. Stress and emotional wellbeing of parents due to change in routine for children with Autism Spectrum Disorder (ASD) at home during COVID-19 pandemic in Saudi Arabia. Res Dev Disabil. 2021 Jan;108:103822.

14.	Amirian H, Solimani S, Maleki F, Ghasemi SR, Reshadat S, Gilan NR. A Study on Health-Related Quality of Life, Depression, and Associated Factors Among Parents of Children with Autism in Kermanshah, Iran. Iran J Psychiatry Behav Sci. 2017 June; 11(2):e7832.

15.	Anixt JS, Murray DS, Coury DL, Kuhlthau KA, Eskra D, Seide J, et al. Improving Behavior Challenges and Quality of Life in the Autism Learning Health Network. Pediatrics. 2020 Apr;145(Suppl 1):S20–9.

16.	Asahar SF, Malek KA, Isa MR. Quality of Life and Child’s Autism-Specific Difficulties among Malaysian Main Caregivers: A Cross-Sectional Study. Int J Environ Res Public Health. 2021 Sept 18;18(18):9861.

17.	Barbosa MRP, Fernandes FDM. Qualidade de vida dos cuidadores de crianças com transtorno do espectro autístico. Rev Soc Bras Fonoaudiol. 2009;14:482–6.

18.	Beheshti SZ, Hosseini SS, Maroufizadeh S, Almasi-Hashiani A. Occupational performance of children with autism spectrum disorder and quality of life of their mothers. BMC Res Notes. 2022 Jan 15;15(1):18.

19.	Benjak T. Subjective Quality of Life for Parents of Children with Autism Spectrum Disorders in Croatia. Applied Research Quality Life. 2011 Mar 1;6(1):91–102.

20.	Bent S, Wahlberg J, Chen Y, Widjaja F, McDonald MG, Hendren RL. Quality of Life Among School-Age Children With Autism: The Oak Hill School Outcomes Study. Semin Pediatr Neurol. 2020 July;34:100808.

21.	Biscaldi M, Paschke-Müller M, Rauh R, Schaller UM. [Evaluation of the Freiburg TOMTASS: A theory-of-mind based social skills group training for children and adolescents with a high-functioning autism spectrum disorder]. Zeitschrift für Psychiatrie, Psychologie und Psychotherapie. 2016 Sept;64(4):269–75.

22.	Broquere M, Soussana M, Michelon C, Rattaz C, Brisot J, Baghdadli A. [Impact of anxiety disorders on quality of life of adolescents with autism spectrum disorder without intellectual disability]. Encephale. 2016 Dec;42(6):499–505.

23.	Bulkley JE, Varga AM, Dickerson JF, Crawford P, Croen LA, Daida YG, et al. A framework for measuring the cost to families of caring for children’s health: the design, methodology, and study population of the r-Kids study. BMC Pediatr. 2023 Mar 20;23(1):128.

24.	Cappe E, Downes N, Albert-Benaroya S, Ech-Chouikh JA, De Gaulmyn A, Luperto L, et al. Preliminary Results of the Effects of a Psychoeducational Program on Stress and Quality of Life Among French Parents of an Child With Autism. Focus Autism Other Dev Disabl. 2021 Sept 1;36(3):176–86.

25.	Cardelle-Pérez F, Banacloig-Delgado C, García-García L. Resilience and family quality of life in parents of children with autism spectrum disorders. Clin Child Psychol Psychiatry. 2024 Jan;29(1):245–58.

26.	Celik H, Acikel SB, Ozdemir MAF, Aksoy E, Oztoprak U, Ceylan N, et al. Evaluation of the clinical characteristics of children with autism spectrum disorder and epilepsy and the perception of their parents on quality of life. Epilepsy Res. 2021 May;172:106599.

27.	Cerit E, Şahin E, Düz S, Karaçul FE, Özdurak Singin RH. Effect of Therapeutic Recreation on Mothers Having Children with Autism Spectrum Disorders. Eur J Mol Clin Med. 2022;9(7):6087–100.

28.	Chaabène I, Halayem S, Mrabet A, Hajri M, Bouden A. Quality of live among parents of children with autism spectrum disorders in Tunisia. Tunis Med. 2018 Mar;96(3):172–7.

29.	Chezan LC, Liu J, Cholewicki JM, Drasgow E, Ding R, Warman A. A Psychometric Evaluation of the Quality of Life for Children with Autism Spectrum Disorder Scale. J Autism Dev Disord. 2022 Apr;52(4):1536–52.

30.	Chezan LC, Liu J, Drasgow E, Ding R, Magana A. The Quality of Life for Children with Autism Spectrum Disorder Scale: Factor Analysis, MIMIC Modeling, and Cut-Off Score Analysis. J Autism Dev Disord. 2023 Aug;53(8):3230–45.

31.	Chiang HM. A Parent Education Program for Parents of Chinese American Children With Autism Spectrum Disorders (ASDs): A Pilot Study. Focus Autism Other Dev Disabl. 2014 June 1;29(2):88–94.

32.	Chuang IC, Tseng MH, Lu L, Shieh JY, Cermak SA. Predictors of the health-related quality of life in preschool children with Autism spectrum disorders. Research in Autism Spectrum Disorders. 2014 Sept;8(9):1062–70.

33.	Clark BG, Magill-Evans JE, Koning CJ. Youth With Autism Spectrum Disorders: Self- and Proxy-Reported Quality of Life and Adaptive Functioning. Focus Autism Other Dev Disabl. 2015 Mar 1;30(1):57–64.

34.	Clark M, Dissanayake C. Characteristics of children attending an autism specific early learning and care setting prior to and following introduction of the National Disability Insurance Scheme. J Intellect Dev Disabil. 2024 Mar;49(1):36–45.

35.	Coales C, Heaney N, Ricketts J, Dockrell JE, Lindsay G, Palikara O, et al. Health-related quality of life in children with autism spectrum disorders and children with developmental language disorders. Autism & Developmental Language Impairments. 2019 Jan 1;4:2396941519851225.

36.	Dai Y, Chen M, Deng T, Huang B, Ji Y, Feng Y, et al. The importance of parenting self-efficacy and social support for family quality of life in children newly diagnosed with autism spectrum disorder: A one-year follow-up study. Autism Res. 2024 Jan;17(1):148–61.

37.	Dardas LA, Ahmad MM. Predictors of quality of life for fathers and mothers of children with autistic disorder. Res Dev Disabil. 2014 June;35(6):1326–33.

38.	Dardas LA, Ahmad MM. Psychosocial correlates of parenting a child with autistic disorder. J Nurs Res. 2014 Sept;22(3):183–91.

39.	Dardas LA, Ahmad MM. Quality of life among parents of children with autistic disorder: a sample from the Arab world. Res Dev Disabil. 2014 Feb;35(2):278–87.

40.	Dardas LA, Ahmad MM. Validation of the World Health Organization’s Quality of Life Questionnaire with parents of children with autistic disorder. J Autism Dev Disord. 2014 Sept;44(9):2257–63.

41.	Dardas LA, Ahmad MM. Coping strategies as mediators and moderators between stress and quality of life among parents of children with autistic disorder. Stress Health. 2015 Feb;31(1):5–12.

42.	Dardas LA, Ahmad MM. For fathers raising children with autism, do coping strategies mediate or moderate the relationship between parenting stress and quality of life? Res Dev Disabil. 2015 Jan;36C:620–9.

43.	Davy G, Barbaro J, Unwin K, Clark M, Jellett R, Date P, et al. Leisure, community, workforce participation and quality of life in primary and secondary caregivers of autistic children. Autism Res. 2024 Apr;17(4):799–811.

44.	De Castro GG, Vieira ALS, Santos NM de F. [Factors associated with the quality of life of mothers of patients with Autism Spectrum Disorder]. O Mundo da Saúde. 2022 May 1;46:043–53.

45.	Delahaye J, Kovacs E, Sikora D, Hall TA, Orlich F, Clemons TE, et al. The relationship between Health-Related Quality of Life and sleep problems in children with Autism Spectrum Disorders. Research in Autism Spectrum Disorders. 2014 Mar 1;8(3):292–303.

46.	Derguy C, Roux S, Portex M, M’bailara K. An ecological exploration of individual, family, and environmental contributions to parental quality of life in autism. Psychiatry Res. 2018 Oct;268:87–93.

47.	Drüsedau L, Schoba A, Conzelmann A, Sokolov A, Hautzinger M, Renner TJ, et al. A structured group intervention (TüTASS) with focus on self-perception and mindfulness for children with autism spectrum disorder, ASD. A pilot study. Eur Arch Psychiatry Clin Neurosci. 2022 Mar;272(2):177–85.

48.	Domellöf E, Hedlund L, Ödman P. Health-related quality of life of children and adolescents with functional disabilities in a northern Swedish county. Qual Life Res. 2014 Aug;23(6):1877–82.

49.	Due C, Goodwin Smith I, Allen P, Button E, Cheek C, Quarmby L, et al. A pilot study of social inclusion and quality of life for parents of children with autism spectrum disorder. Journal of Intellectual & Developmental Disability. 2018 Jan 2;43(1):73–82.

50.	Durhan TA, Türkmen E, Karaman M, Suveren C. Quality of Life in Parents of Children With Autism. International Journal of Religion. 2024;5(4):75–81.

51.	Eapen V, Crnčec R, Walter A, Tay KP. Conceptualisation and development of a quality of life measure for parents of children with autism spectrum disorder. Autism Res Treat. 2014;2014:160783.

52.	Eapen V, Islam R, Azim SI, Masi A, Klein L, Karlov L. Factors Impacting Parental Quality of Life in Preschool Children on the Autism Spectrum. J Autism Dev Disord. 2024 Mar;54(3):943–54.

53.	Eapen V, Karlov L, John JR, Beneytez C, Grimes PZ, Kang YQ, et al. Quality of life in parents of autistic children: A transcultural perspective. Front Psychol. 2023 Feb 23;14:1022094.

54.	Egilson ST, Ólafsdóttir LB, Leósdóttir T, Saemundsen E. Quality of life of high-functioning children and youth with autism spectrum disorder and typically developing peers: Self- and proxy-reports. Autism. 2017 Feb;21(2):133–41.

55.	Elias AV, Assumpção Jr FB. Qualidade de vida e autismo. Arq Neuro-Psiquiatr. 2006;64:295–9.

56.	Eskow K, Pineles L, Summers JA. Exploring the Effect of Autism Waiver Services on Family Outcomes. Journal of Policy and Practice in Intellectual Disabilities. 2011;8(1):28–35.

57.	Eskow K, Ann Summers J, Chasson GS, Mitchell R. The Association Between Family–Teacher Partnership Satisfaction and Outcomes of Academic Progress and Quality of Life for Children/Youth With Autism. Journal of Policy and Practice in Intellectual Disabilities. 2018;15(1):16–25.

58.	Fávero-Nunes MÂ, Santos MA dos. Depression and quality of life in mothers of children with pervasive developmental disorders. Rev Latino-Am Enfermagem. 2010;18:33–40.

59.	Fayız AN, Palancı M. Investigation of Self-Efficacy, Quality of Life Perceptions and Resilience Levels of Parents with Children with Autism by Mediator Variable Analysis. Education and Science. 2023 Apr 4;48(214):73–98.

60.	Feng Y, Zhou X, Qin X, Cai G, Lin Y, Pang Y, et al. Parental self-efficacy and family quality of life in parents of children with autism spectrum disorder in China: The possible mediating role of social support. J Pediatr Nurs. 2022;63:159–67.

61.	Flor J, Bellando J, Lopez M, Shui A. Developmental functioning and medical Co-morbidity profile of children with complex and essential autism. Autism Res. 2017 Aug;10(8):1344–52.

62.	Forouzanfar S, Babakhani N, Sodagar S. Health Related Quality of Life among Mothers of Children with Autism Spectrum Disorder and Its Predictors. Indian Journal of Public Health Research & Development. 2016; 7(2).

63.	Fourcade C, Kruck J, Rogé B. [Parents facing the Handicap of their Children affected by Autism Spectrum Disorder: Assessment of Caregiver Strain, Resilience and Quality of Life.]. ANAE Approche Neuropsychologique des Apprentissages chez l’Enfant. 2015;27(3):365–75.

64.	Gabra RH, Hashem DF, Ahmed GK. The possible relation between stigma, parent psychiatric symptoms, quality of life and the disease burden in families of children with autism spectrum disorder in Egypt: a multicentre study. The Egyptian Journal of Neurology, Psychiatry and Neurosurgery. 2021 Dec 13;57(1):170.

65.	Gabra RH, Hashem DF. Comparison of caregivers’ characteristics, stigma, and disease burden of children with autism spectrum disorder and attention-deficit disorder in Egypt. Middle East Current Psychiatry. 2021 Dec 28;28(1):83.

66.	Gardiner E, Iarocci G. Family Quality of Life and ASD: The Role of Child Adaptive Functioning and Behavior Problems. Autism Research. 2015;8(2):199–213.

67.	Garrido D, Carballo G, Ortega E, García-Retamero R. [Psychological adaptation in children with autism spectrum disorder and its effect on family quality of life]. Rev Neurol. 2020 Aug 16;71(4):127–33.

68.	Gengoux GW, Schapp S, Burton S, Ardel CM, Libove RA, Baldi G, et al. Effects of a parent-implemented Developmental Reciprocity Treatment Program for children with autism spectrum disorder. Autism. 2019 Apr;23(3):713–25.

69.	Green CC, Smith J, Bent CA, Chetcuti L, Sulek R, Uljarević M, et al. Differential predictors of well-being versus mental health among parents of pre-schoolers with autism. Autism. 2021 May;25(4):1125–36.

70.	Green J, Leadbitter K, Ellis C, Taylor L, Moore HL, Carruthers S, et al. Combined social communication therapy at home and in education for young autistic children in England (PACT-G): a parallel, single-blind, randomised controlled trial. Lancet Psychiatry. 2022 Apr;9(4):307–20.

71.	Günal A, Bumin G, Huri M. The Effects of Motor and Cognitive Impairments on Daily Living Activities and Quality of Life in Children with Autism. Journal of Occupational Therapy, Schools, & Early Intervention. 2019 Oct 2;12(4):444–54.

72.	Gunes S, Ekinci O. Health-related quality of life in children and adolescent with ASD: the role of mothers’ emotional symptoms. AP. 2019 Oct 1;20(5):548–55.

73.	Hand BN, Lane AE, De Boeck P, Basso DM, Nichols-Larsen DS, Darragh AR. Caregiver Burden Varies by Sensory Subtypes and Sensory Dimension Scores of Children with Autism. J Autism Dev Disord. 2018 Apr;48(4):1133–46.

74.	Hatfield M, Falkmer M, Falkmer T, Ciccarelli M. Effectiveness of the BOOST-ATM online transition planning program for adolescents on the autism spectrum: a quasi-randomized controlled trial. Child Adolesc Psychiatry Ment Health. 2017;11:54.

75.	Hedgecock JB, Dannemiller LA, Shui AM, Rapport MJ, Katz T. Associations of Gross Motor Delay, Behavior, and Quality of Life in Young Children With Autism Spectrum Disorder. Phys Ther. 2018 Apr 1;98(4):251–9.

76.	Herpers PCM, Klip H, Rommelse NNJ, Greven CU, Buitelaar JK. Associations between high callous-unemotional traits and quality of life across youths with non-conduct disorder diagnoses. Eur Child Adolesc Psychiatry. 2016 May;25(5):547–55.

77.	Herrera E, Baena S, Hidalgo V, Trigo E. The relationship between family quality of life, mindful attention, and social support in families of people with autism spectrum disorder. Int J Dev Disabil. 2024;70(4):604–14.

78.	Hoefman R, Payakachat N, van Exel J, Kuhlthau K, Kovacs E, Pyne J, et al. Caring for a child with autism spectrum disorder and parents’ quality of life: application of the CarerQol. J Autism Dev Disord. 2014 Aug;44(8):1933–45.

79.	Howe SJ, Baraskewich J, McMorris CA. Anxiety in Autistic Youth: Understanding the Impact on Child, Caregiver, and Family Quality of Life. J Child Fam Stud. 2023 Mar 1;32(3):892–907.

80.	Ismail MF, Safii R, Saimon R, Rahman MM. Quality of Life Among Malaysian Parents with Autism Spectrum Disorder Child: The Double ABCX Model Approach. J Autism Dev Disord. 2022 Jan;52(1):113–23.

81.	Jahan MU, Karim MR, Rahman A, Akhter S. Cognitive Functions and Health Related Quality of Life of Institutional Autism Spectrum Disorder Children in Dhaka city. Bangladesh Med Res Counc Bull. 2015 Dec;41(3):151–9.

82.	Jain A, Ahmed N, Mahour P, Agarwal V, Shrivastav NK, Chandrakanta. Quality of Life of Caregivers of Autistic Children and Adolescents Visiting Health Facilities in Lucknow City, Uttar Pradesh, India: A Cross-sectional Study. Indian J Community Med. 2019;44(2):157–61.

83.	Jamison TR, Schuttler JO. Examining social competence, self-perception, quality of life, and internalizing and externalizing symptoms in adolescent females with and without autism spectrum disorder: a quantitative design including between-groups and correlational analyses. Mol Autism. 2015;6:53.

84.	Ji B, Sun M, Yi R, Tang S. Multidisciplinary parent education for caregivers of children with autism spectrum disorders. Arch Psychiatr Nurs. 2014 Oct;28(5):319–26.

85.	Ji B, Zhao I, Turner C, Sun M, Yi R, Tang S. Predictors of health-related quality of life in Chinese caregivers of children with autism spectrum disorders: a cross-sectional study. Arch Psychiatr Nurs. 2014 Oct;28(5):327–32.

86.	Jones S, Bremer E, Lloyd M. Autism spectrum disorder: family quality of life while waiting for intervention services. Qual Life Res. 2017 Feb;26(2):331–42.

87.	Katsiana A, Strimpakos N, Ioannis V, Kapreli E, Sofologi M, Bonti E, et al. Health-related Quality of Life in Children with Autism Spectrum Disorder and Children with Down Syndrome. Mater Sociomed. 2020 June;32(2):93–8.

88.	Khanna R, Madhavan SS, Smith MJ, Patrick JH, Tworek C, Becker-Cottrill B. Assessment of health-related quality of life among primary caregivers of children with autism spectrum disorders. J Autism Dev Disord. 2011 Sept;41(9):1214–27.

89.	Khanna R, Madhavan SS, Smith MJ, Tworek C, Patrick JH, Becker-Cottrill B. Psychometric properties of the Caregiver Strain Questionnaire (CGSQ) among caregivers of children with autism. Autism. 2012 Mar;16(2):179–99.

90.	Kheir NM, Ghoneim OM, Sandridge AL, Hayder SA, Al-Ismail MS, Al-Rawi F. Concerns and considerations among caregivers of a child with autism in Qatar. BMC Research Notes. 2012 June 13;5(1):290.

91.	Kheir N, Ghoneim O, Sandridge AL, Al-Ismail M, Hayder S, Al-Rawi F. Quality of life of caregivers of children with autism in Qatar. Autism. 2012 May;16(3):293–8.

92.	Kilincaslan A, Kocas S, Bozkurt S, Kaya I, Derin S, Aydin R. Daily living skills in children with autism spectrum disorder and intellectual disability: A comparative study from Turkey. Res Dev Disabil. 2019 Feb;85:187–96.

93.	Kim Y, Kim M, Park C, You JSH. Effects of Integrative Autism Therapy on Multiple Physical, Sensory, Cognitive, and Social Integration Domains in Children and Adolescents with Autism Spectrum Disorder: A 4-Week Follow-Up Study. Children (Basel). 2022 Dec 15;9(12):1971.

94.	Calisan Kinter R, Ozbaran B, Inal Kaleli I, Kose S, Bildik T, Ghaziuddin M. The Sensory Profiles, Eating Behaviors, and Quality of Life of Children with Autism Spectrum Disorder and Avoidant/Restrictive Food Intake Disorder. Psychiatr Q. 2024 Mar;95(1):85–106.

95.	Kose S, Erermis S, Ozturk O, Ozbaran B, Demiral N, Bildik T, et al. Health Related Quality of Life in children with Autism Spectrum Disorders: The clinical and demographic related factors in Turkey. Research in Autism Spectrum Disorders. 2013 Feb 1;7(2):213–20.

96.	Kousha M, Attar HA, Shoar Z. Anxiety, depression, and quality of life in Iranian mothers of children with autism spectrum disorder. J Child Health Care. 2016 Sept;20(3):405–14.

97.	Kousha M, Soleimani R, Masjedi M, Hoshyarfard S, Yhayazadeh A. A comparison of mental health, quality of life, and happiness in mothers of children with autism spectrum disorders versus mothers with healthy children. International Journal of Pharmaceutical Sciences and Research. 2019;10(2):856–62.

98.	Kuhlthau K, Orlich F, Hall TA, Sikora D, Kovacs EA, Delahaye J, et al. Health-Related Quality of Life in children with autism spectrum disorders: results from the autism treatment network. J Autism Dev Disord. 2010 June;40(6):721–9.

99.	Kuhlthau K, Kovacs E, Hall T, Clemmons T, Orlich F, Delahaye J, et al. Health-related quality of life for children with ASD: Associations with behavioral characteristics. Research in Autism Spectrum Disorders. 2013 Sept 1;7(9):1035–42.

100.	Kuhlthau K, Payakachat N, Delahaye J, Hurson J, Pyne JM, Kovacs E, et al. Quality of life for parents of children with autism spectrum disorders. Research in Autism Spectrum Disorders. 2014 Oct 1;8(10):1339–50.

101.	Kuhlthau KA, McDonnell E, Coury DL, Payakachat N, Macklin E. Associations of quality of life with health-related characteristics among children with autism. Autism. 2018 Oct;22(7):804–13.

102.	Kuru N, Piyal B. Perceived social support and quality of life of parents of children with Autism. Niger J Clin Pract. 2018 Sept;21(9):1182–9.

103.	Lal DV, Sowmi S, Ambikapathy BS. PedsQL-4.0 in children with Autism Spectrum Disorder. International Journal of Research in Pharmaceutical Sciences. 2020;11(2):171–4.

104.	Lanning BA, Baier MEM, Ivey-Hatz J, Krenek N, Tubbs JD. Effects of equine assisted activities on autism spectrum disorder. J Autism Dev Disord. 2014 Aug;44(8):1897–907.

105.	Lanyi J, Mannion A, Chen JL, Leader G. Relationship between Comorbid Psychopathology in Children and Adolescents with Autism Spectrum Disorder and Parental Well-being. Dev Neurorehabil. 2022 Apr;25(3):151–61.

106.	Lara EB, de los Pinos M del C. Family assessment methods for school children with autism spectrum disorders. Opción. 2015;31(2):122–43.

107.	Leader G, O’Reilly M, Gilroy SP, Chen JL, Ferrari C, Mannion A. Comorbid Feeding and Gastrointestinal Symptoms, Challenging Behavior, Sensory Issues, Adaptive Functioning and Quality of Life in Children and Adolescents with Autism Spectrum Disorder. Dev Neurorehabil. 2021 Jan;24(1):35–44.

108.	Leader G, Flynn C, O’Rourke N, Coyne R, Caher A, Mannion A. Comorbid Psychopathology, Challenging Behavior, Sensory Issues, Adaptive Behavior and Quality of Life in Children and Adolescents with Autism Spectrum Disorder. Dev Neurorehabil. 2021 Aug;24(6):397–407.

109.	Leader G, Glynn C, Kirkpatrick B, Chen JL, O’Súilleabháin PS, Mannion A. Familial sleep and autism spectrum disorder: a pilot actigraphy study of sleep quality, quality of life and psychological distress. Ir J Psychol Med. 2022 Sept;39(3):261–71.

110.	Leader G, Dooley E, Whelan S, Gilroy SP, Chen JL, Farren Barton A, et al. Attention-Deficit/Hyperactivity Disorder Symptoms, Gastrointestinal Symptoms, Sleep Problems, Challenging Behavior, Adaptive Behavior, and Quality of Life in Children and Adolescents with Autism Spectrum Disorder. Dev Neurorehabil. 2022 May;25(4):217–28.

111.	Lee GK, Lopata C, Volker MA, Thomeer ML, Nida RE, Toomey JA, et al. Health-Related Quality of Life of Parents of Children With High-Functioning Autism Spectrum Disorders. Focus Autism Other Dev Disabl. 2009 Dec 1;24(4):227–39.

112.	Lee GK, Lopata C, Volker MA, Thomeer ML, Toomey JA, Rodgers JD, et al. Stress, Resiliency Factors, Quality of Life Among Caregivers of Children with High Functioning Autism Spectrum Disorders (HFASDs). The Australian Journal of Rehabilitation Counselling. 2012 July;18(1):25–36.

113.	Lee MH, Min A, Park C, Kim I. How Do Sleep Disturbances Relate to Daytime Functions, Care-related Quality of Life, and Parenting Interactions in Mothers of Children with Autism Spectrum Disorder? J Autism Dev Disord. 2023 July;53(7):2764–72.

114.	Lee GK, Shivers CM. Factors that affect the physical and mental health of caregivers of school-age children and transitioning young adults with autism spectrum disorder. J Appl Res Intellect Disabil. 2019 May;32(3):622–34.

115.	Lei X, Kantor J. Social support and family quality of life in Chinese families of children with autism spectrum disorder: the mediating role of family cohesion and adaptability. Int J Dev Disabil. 2022;68(4):454–61.

116.	Lei X, Kantor J. Correlates of Social Support and Family Quality of Life in Chinese Caregivers of Children with Autism Spectrum Disorder. International Journal of Disability, Development and Education. 2023 Sept 19;70(6):963–76.

117.	Leonard H, Whitehouse A, Jacoby P, Benke T, Demarest S, Saldaris J, et al. Quality of life beyond diagnosis in intellectual disability - Latent profiling. Res Dev Disabil. 2022 Oct;129:104322.

118.	Lichtlé J, Devouche E, Dialahy IZ, de Gaulmyn A, Amestoy A, Coutelle R, et al. Development, psychometric evaluation, and factor analysis of an instrument measuring quality of life in autistic preschoolers. Int J Methods Psychiatr Res. 2023 Dec 22;33(1):e2002.

119.	Likhitweerawong N, Boonchooduang N, Louthrenoo O. Parenting Styles, Parental Stress, and Quality of Life Among Caregivers of Thai Children with Autism. International Journal of Disability, Development and Education. 2022 Nov 2;69(6):2094–107.

120.	Logrieco MG, Casula L, Ciuffreda GN, Novello RL, Spinelli M, Lionetti F, et al. Risk and protective factors of quality of life for children with autism spectrum disorder and their families during the COVID-19 lockdown. An Italian study. Res Dev Disabil. 2022 Jan;120:104130.

121.	Lopez-Espejo MA, Nuñez AC, Moscoso OC, Escobar RG. Brief Report: Health-Related Quality of Life in Preschoolers with Autism Spectrum Disorder is Related to Diagnostic Age and Autistic Symptom Severity. J Autism Dev Disord. 2021 Dec;51(12):4704–10.

122.	Lopez-Espejo MA, Nuñez AC, Ruz M, Saez V, Moscoso OC, Escobar RG. Caregiver-Child Agreement in Health-Related Quality of Life of Autistic Children and Adolescents. J Autism Dev Disord. 2023 Mar;53(3):977–86.

123.	Losada-Puente L, Baña M, Asorey MJF. Family quality of life and autism spectrum disorder: Comparative diagnosis of needs and impact on family life. Res Dev Disabil. 2022 May;124:104211.

124.	Mahjoob M, Cardy R, Penner M, Anagnostou E, Andrade BF, Crosbie J, et al. Predictors of health-related quality of life for children with neurodevelopmental conditions. Sci Rep. 2024 Mar 16;14(1):6377.

125.	Mannion A, Leader G. Relationship between child sleep problems in autism spectrum disorder and parent mental health and well-being. Sleep Med. 2023 Sept;109:4–10.

126.	Martin CA, Sciberras E, Papadopoulos N, Engel L, Hiscock H, Williams K, et al. Associations Between Child Sleep Problem Severity and Maternal Well-Being in Children with Autism Spectrum Disorder. J Autism Dev Disord. 2021 July;51(7):2500–10.

127.	Masuri MG, Shafie WNW, Dahlan A, Ibrahim SAS, Thanapalan CKK, Daud AZC, et al. Emotional States and Quality of Life among Parents of Children with Autism Spectrum Disorder (ASD) during COVID-19 in Malaysia. Journal of Health and Translational Medicine (JUMMEC). 2023 Sept 15;120–32.

128.	May T, Brignell A, Williams K. Autism Spectrum Disorder Prevalence in Children Aged 12-13 Years From the Longitudinal Study of Australian Children. Autism Res. 2020 May;13(5):821–7.

129.	Mazon C, Fage C, Consel C, Amestoy A, Hesling I, Bouvard M, et al. Cognitive Mediators of School-Related Socio-Adaptive Behaviors in ASD and Intellectual Disability Pre- and Adolescents: A Pilot-Study in French Special Education Classrooms. Brain Sci. 2019 Nov 21;9(12):334.

130.	McAuliffe T, Cordier R, Vaz S, Thomas Y, Falkmer T. Quality of Life, Coping Styles, Stress Levels, and Time Use in Mothers of Children with Autism Spectrum Disorders: Comparing Single Versus Coupled Households. J Autism Dev Disord. 2017 Oct;47(10):3189–203.

131.	McStay RL, Trembath D, Dissanayake C. Maternal stress and family quality of life in response to raising a child with autism: from preschool to adolescence. Res Dev Disabil. 2014 Nov;35(11):3119–30.

132.	McStay RL, Dissanayake C, Scheeren A, Koot HM, Begeer S. Parenting stress and autism: the role of age, autism severity, quality of life and problem behaviour of children and adolescents with autism. Autism. 2014 July;18(5):502–10.

133.	McStay RL, Trembath D, Dissanayake C. Stress and family quality of life in parents of children with autism spectrum disorder: parent gender and the double ABCX model. J Autism Dev Disord. 2014 Dec;44(12):3101–18.

134.	Mello C, Rivard M, Terroux A, Mercier C. Quality of Life in Families of Young Children With Autism Spectrum Disorder. Am J Intellect Dev Disabil. 2019 Nov;124(6):535–48.

135.	Menezes M, Mazurek MO. Associations between domains of health-related quality of life and comorbid emotional and behavioral problems in youth with autism spectrum disorder. Res Autism Spectr Disord. 2021 Apr;82:101740.

136.	Meral BF, Cavkaytar A, Turnbull AP, Wang M. Family Quality of Life of Turkish Families Who Have Children with Intellectual Disabilities and Autism. Research and Practice for Persons with Severe Disabilities. 2013 Dec 1;38(4):233–46.

137.	Millau M, Rivard M, Mello C. Quality of Life in Immigrant Parents of Children With Autism Spectrum Disorder: A Comparison With Parents From the Host Culture. J Child Fam Stud. 2019 June 1;28(6):1512–23.

138.	Moretto G, Ishihara M, Ribeiro M, Caetano SC, Perissinoto J, Tamanaha AC. Interference of the communicative profile of children with autism spectrum disorders upon their mother’s quality of life. Codas. 2020;32(6):e20190170.

139.	Musetti A, Zagaria A, Pezzi M, Fante C, Dioni B, Raffin C, et al. Parental quality of life, child adjustment and adult attachment in parents of children and adolescents with Autism Spectrum Disorder. Res Dev Disabil. 2024 Mar;146:104684.

140.	Naheed A, Islam MS, Hossain SW, Ahmed HU, Uddin MMJ, Tofail F, et al. Burden of major depressive disorder and quality of life among mothers of children with autism spectrum disorder in urban bangladesh. Autism Res. 2020 Feb;13(2):284–97.

141.	Naheed A, Islam MS, Brooks MB, Fawzi MCS, Ashraf MN, Ahmed HU, et al. Feasibility of a school-based mental health program implementation to improve the status of depression and quality of life of mothers of children with autism spectrum disorders in urban Bangladesh: MENTHOL study. Glob Ment Health (Camb). 2022;9:146–56.

142.	Nuske HJ, Hedley D, Tseng CH, Begeer S, Dissanayake C. Emotion Regulation Strategies in Preschoolers with Autism: Associations with Parent Quality of Life and Family Functioning. J Autism Dev Disord. 2018 Apr;48(4):1287–300.

143.	Oakley BF, Tillmann J, Ahmad J, Crawley D, San José Cáceres A, Holt R, et al. How do core autism traits and associated symptoms relate to quality of life? Findings from the Longitudinal European Autism Project. Autism. 2021 Feb;25(2):389–404.

144.	Öz B, Yüksel T, Nasiroğlu S. Depression-Anxiety Symptoms and Stigma Perception in Mothers of Children with Autism Spectrum Disorder. Noro Psikiyatr Ars. 2019 Nov 28;57(1):50–5.

145.	Ozboke C, Yanardag M, Yilmaz I. Exploring the relationships between motor proficiency, independence and quality of life in adolescents with autism spectrum disorder. Int J Dev Disabil. 2022;68(6):850–7.

146.	Özgür BG, Aksu H, Eser E. Factors affecting quality of life of caregivers of children diagnosed with autism spectrum disorder. Indian J Psychiatry. 2018;60(3):278–85.

147.	Ozturk O, Erermis S, Ercan ES, Gulen F, Basay BK, Basay O, et al. Does the quality of life in autism spectrum disorder differ from other chronic disorders and healthy children? AP. 2016 Oct 1;17(5):419–26.

148.	Palmer JK, van der Pols JC, Sullivan KA, Staudacher HM, Byrne R. A Double-Blind Randomised Controlled Trial of Prebiotic Supplementation in Children with Autism: Effects on Parental Quality of Life, Child Behaviour, Gastrointestinal Symptoms, and the Microbiome. J Autism Dev Disord. 2025 Mar;55(3):775–88.

149.	Papadopoulos A, Siafaka V, Tsapara A, Tafiadis D, Kotsis K, Skapinakis P, et al. Measuring parental stress, illness perceptions, coping and quality of life in families of children newly diagnosed with autism spectrum disorder. BJPsych Open. 2023 May 18;9(3):e84.

150.	Parsons D, Cordier R, Lee H, Falkmer T, Vaz S. Stress, coping, and quality of life in families with a child with ASD living regionally. Journal of Child and Family Studies. 2020;29(2):546–58.

151.	Patel AD, Arya A, Agarwal V, Gupta PK, Agarwal M. Burden of care and quality of life in caregivers of children and adolescents with autism spectrum disorder. Asian J Psychiatr. 2022 Apr;70:103030.

152.	Pavlova I, Bodnar I, Hamade A, Mosler D, Małolepszy E, Drozdek-Małolepsza T. Relation between quality of life and physical literacy of young adolescents with Autism Spectrum Disorder. International Journal of Special Education. 2021;36(2):13–26.

153.	Pecor KW, Barbayannis G, Yang M, Johnson J, Materasso S, Borda M, et al. Quality of Life Changes during the COVID-19 Pandemic for Caregivers of Children with ADHD and/or ASD. Int J Environ Res Public Health. 2021 Apr 1;18(7):3667.

154.	Perumal V, Veeraraghavan V, Lekhra OP. Quality of life in families of children with autism spectrum disorder in India. Journal of Pharmacy Research. 2014;8(6):791–7.

155.	Pisula E, Porębowicz-Dörsmann A. Family functioning, parenting stress and quality of life in mothers and fathers of Polish children with high functioning autism or Asperger syndrome. PLoS One. 2017;12(10):e0186536.

156.	Poirier N, Belzil A, Cappe É. [Quality of life of mothers of children with autism spectrum disorder attending special classes in primary or secondary education.]. Journal de Thérapie Comportementale et Cognitive. 2018;28(3):141–51.

157.	Potvin MC, Snider L, Prelock PA, Wood-Dauphinee S, Kehayia E. Health-related quality of life in children with high-functioning autism. Autism. 2015 Jan;19(1):14–9.

158.	Prasitwut P, Wantanakorn P, Chuchottaworn K, Reangkanjanaseart S, Chuthapisith J. Effectiveness of Fabric Weaving Therapy for Children with Autism Spectrum Disorder: A Randomized Waitlist-Controlled Trial. J Integr Complement Med. 2024 Mar;30(3):261–8.

159.	Pratesi CB, Garcia AB, Pratesi R, Gandolfi L, Hecht M, Nakano EY, et al. Quality of Life in Caregivers of Children and Adolescents with Autistic Spectrum Disorder: Development and Validation of the Questionnaire. Brain Sci. 2021 July 13;11(7):924.

160.	Raju S, Hepsibah PEV, Niharika MK. Quality of life in parents of children with Autism spectrum disorder: Emphasizing challenges in the Indian context. Int J Dev Disabil. 2023;69(3):371–8.

161.	Ran Q, Tu L, Wu Y, Zhang S, Zhang E, Li H, et al. Formal social support and quality of life of caregivers with autistic children: a large-scale nationwide survey in China. Front Public Health. 2023;11:1282778.

162.	Randall M, Sciberras E, Brignell A, Ihsen E, Efron D, Dissanayake C, et al. Autism spectrum disorder: Presentation and prevalence in a nationally representative Australian sample. Aust N Z J Psychiatry. 2016 Mar;50(3):243–53.

163.	Rayan A, Ahmad M. Effectiveness of mindfulness-based interventions on quality of life and positive reappraisal coping among parents of children with autism spectrum disorder. Res Dev Disabil. 2016 Aug;55:185–96.

164.	Renford NGR, Arulappan J, Thomas KDE, Karuppiah KM, Chinnathambi K, Thangaswamy GC. Quality of Life of Caregivers of Children with Autism Spectrum Disorder in Tamil Nadu, South India. International Journal of Nutrition, Pharmacology, Neurological Diseases. 2020 June;10(2):57.

165.	Rizk S, Pizur-Barnekow K, Darragh AR. Leisure and Social Participation and Health-Related Quality of Life in Caregivers of Children with Autism. OTJR: Occupational Therapy Journal of Research. 2011 Oct 1;31(4):164–71.

166.	Roberts J, Williams K, Carter M, Evans D, Parmenter T, Silove N, et al. A randomised controlled trial of two early intervention programs for young children with autism: Centre-based with parent program and home-based. Research in Autism Spectrum Disorders. 2011 Oct 1;5(4):1553–66.

167.	Roberts CA, Smith KC, Sherman AK. Comparison of Online and Face-to-Face Parent Education for Children with Autism and Sleep Problems. J Autism Dev Disord. 2019 Apr;49(4):1410–22.

168.	Robeson M, Hayes K, Shekouh KR, Ahlich E, Zlomke K. Examining daily impact of mood and quality of social support for caregivers of children on the autism spectrum. Research in Autism Spectrum Disorders. 2024 Feb 1;110:102293.

169.	Romaniuk A, Ward M, Henrikson B, Cochrane K, Theule J. Family Quality of Life Perceived by Mothers of Children with ASD and ADHD. Child Psychiatry Hum Dev. 2024 Apr;55(2):510–9.

170.	Russell M, Baldwin CM, McClain D, Matthews N, Smith C, Quan SF. Symptoms of Restless Legs Syndrome in Biological Caregivers of Children with Autism Spectrum Disorders. J Clin Sleep Med. 2017 Jan 15;13(1):105–13.

171.	Russell M, Baldwin CM, Quan SF. Exploring Sleep in Caregivers of Children with Autism Spectrum Disorder (ASD) and the Relationship to Health-Related Quality of Life (HRQoL) and Family Quality of Life (FQoL). Medicina (Kaunas). 2023 Dec 7;59(12):2132.

172.	Saigh BH, Bagadood NH. Screening for health-related quality of life in children and adolescents with an autism spectrum disorder in Saudi Arabia. International Journal of Advanced and Applied Sciences. 2022;9(7):1–8.

173.	Salami S, Alhalal E. Gender differences in predictors of quality of life for parents of children with Autism Spectrum disorder in Saudi Arabia. J Pediatr Nurs. 2024;77:e117–24.

174.	Sari O, Banu H, Yanci A, Bisgin H, Öztürk M. Associations between Physical Activity with Health-Related Quality of Life and Wellbeing among Children with and without Autism. Pakistan Journal of Medical & Health Sciences. 2022;16(3).

175.	Sarol H, Çimen Z. The Effects of Adapted Recreational Physical Activity on the Life Quality of Individuals with Autism. Anthropologist. 2015;21(3): 522-527,

176.	Schertz M, Karni-Visel Y, Tamir A, Genizi J, Roth D. Family quality of life among families with a child who has a severe neurodevelopmental disability: Impact of family and child socio-demographic factors. Res Dev Disabil. 2016;53–54:95–106.

177.	Schlebusch L, Samuels AE, Dada S. South African families raising children with autism spectrum disorders: relationship between family routines, cognitive appraisal and family quality of life. J Intellect Disabil Res. 2016 May;60(5):412–23.

178.	Schlebusch L, Dada S, Samuels AE. Family Quality of Life of South African Families Raising Children with Autism Spectrum Disorder. J Autism Dev Disord. 2017 July;47(7):1966–77.

179.	Schneider S, Clément C, Goltzene MA, Meyer N, Gras-Vincendon A, Schröder CM, et al. Determinants of the evolutions of behaviours, school adjustment and quality of life in autistic children in an adapted school setting: an exploratory study with the International Classification of Functioning, disability and health (ICF). BMC Psychiatry. 2022 May 6;22(1):323.

180.	Selvakumar N, Panicker AS. Stress and Coping Styles in Mothers of Children with Autism Spectrum Disorder. Indian J Psychol Med. 2020 Apr 25;42(3):225–32.

181.	Sheldrick RC, Neger EN, Shipman D, Perrin EC. Quality of life of adolescents with autism spectrum disorders: concordance among adolescents’ self-reports, parents’ reports, and parents’ proxy reports. Qual Life Res. 2012 Feb;21(1):53–7.

182.	Shipman DL, Sheldrick RC, Perrin EC. Quality of life in adolescents with autism spectrum disorders: reliability and validity of self-reports. J Dev Behav Pediatr. 2011;32(2):85–9.

183.	Shu BC. Quality of life of family caregivers of children with autism: The mother’s perspective. Autism. 2009 Jan;13(1):81–91.

184.	Siah PC, Tan SH. Sense of coherence and WHOQoL among parents of children with ASD in Malaysia. International Journal on Disability and Human Development. 2015;14(1):59–66.

185.	Siah PC, Tan SH. Relationships between sense of coherence, coping strategies and quality of life of parents of children with autism in Malaysia: A case study among chinese parents. DCIDJ. 2016;27(1).

186.	Sikora DM, Vora P, Coury DL, Rosenberg D. Attention-deficit/hyperactivity disorder symptoms, adaptive functioning, and quality of life in children with autism spectrum disorder. Pediatrics. 2012 Nov;130 Suppl 2:S91-97.

187.	Soccorso C, Milliken A, Hojlo M, Pawlowski K, Weas S, Sideridis G, et al. Quality of Life and Family Impact in Down Syndrome, Autism Spectrum Disorder, and Co-occurring Down Syndrome and Autism Spectrum Disorder. J Dev Behav Pediatr. 2023 Apr 1;44(3):e185–95.

188.	Sorrenti L, Arena J, Mafodda AV, Filippello P. Perception of Well-Being, Predisposition to Optimism, and Quality of Life of Parents of Children with Autism Spectrum Disorder. Mediterranean Journal of Clinical Psychology. 2021 Aug 8;Vol 9:No 2 (2021).

189.	Stiller A, Stiller C. Parental report of quality of life in autistic children and youths: the role of parents’ (informal) social support, and children’s active leisure time. Int J Dev Disabil. 2024;70(3):354–64.

190.	Süzen İ, Parpucu Tİ, Parpucu HŞ. The Relationship Between Physical Activity Level And Quality of Life of Children with Autism Spectrum Disorder. International Journal of Disabilities Sports &Health Sciences. 2022 June 20;5(1):1–8.

191.	Tait K, Fung F, Hu A, Sweller N, Wang W. Understanding Hong Kong Chinese Families’ Experiences of an Autism/ASD Diagnosis. J Autism Dev Disord. 2016 Apr;46(4):1164–83.

192.	Tavernor L, Barron E, Rodgers J, McConachie H. Finding out what matters: validity of quality of life measurement in young people with ASD. Child Care Health Dev. 2013 July;39(4):592–601.

193.	Taylor LJ, Luk SYL, Leadbitter K, Moore HL, Charman T. Are child autism symptoms, developmental level and adaptive function associated with caregiver feelings of wellbeing and efficacy in the parenting role? Research in Autism Spectrum Disorders. 2021 May 1;83:101738.

194.	Tekinarslan IC. A comparison study of depression and quality of life in Turkish mothers of children with Down syndrome, cerebral palsy, and autism spectrum disorder. Psychol Rep. 2013 Feb;112(1):266–87.

195.	Ten Hoopen LW, de Nijs PFA, Duvekot J, Greaves-Lord K, Hillegers MHJ, Brouwer WBF, et al. Children with an Autism Spectrum Disorder and Their Caregivers: Capturing Health-Related and Care-Related Quality of Life. J Autism Dev Disord. 2020 Jan;50(1):263–77.

196.	Ten Hoopen LW, de Nijs PFA, Duvekot J, Greaves-Lord K, Hillegers MHJ, Brouwer WBF, et al. Caring for Children with an Autism Spectrum Disorder: Factors Associating with Health- and Care-Related Quality of Life of the Caregivers. J Autism Dev Disord. 2022 Nov;52(11):4665–78.

197.	Tilford JM, Payakachat N, Kovacs E, Pyne JM, Brouwer W, Nick TG, et al. Preference-based health-related quality-of-life outcomes in children with autism spectrum disorders: a comparison of generic instruments. Pharmacoeconomics. 2012 Aug 1;30(8):661–79.

198.	Toscano CVA, Carvalho HM, Ferreira JP. Exercise Effects for Children With Autism Spectrum Disorder: Metabolic Health, Autistic Traits, and Quality of Life. Percept Mot Skills. 2018 Feb;125(1):126–46.

199.	Tung LC, Huang CY, Tseng MH, Yen HC, Tsai YP, Lin YC, et al. Correlates of health-related quality of life and the perception of its importance in caregivers of children with Autism. Research in Autism Spectrum Disorders. 2014;8(9):1235–42.

200.	Vahedparast H, Khalafi S, Jahanpour F, Bagherzadeh R. The Actor-Partner Effects of Parenting Stress on Quality of Life Among Parents of Children with ASD: The Mediating Role of Mental Quality of Life. J Autism Dev Disord. 2022 Jan;52(1):142–9.

201.	van Steensel FJA, Bögels SM. CBT for anxiety disorders in children with and without autism spectrum disorders. J Consult Clin Psychol. 2015 June;83(3):512–23.

202.	Varni JW, Handen BL, Corey-Lisle PK, Guo Z, Manos G, Ammerman DK, et al. Effect of aripiprazole 2 to 15 mg/d on health-related quality of life in the treatment of irritability associated with autistic disorder in children: a post hoc analysis of two controlled trials. Clin Ther. 2012 Apr;34(4):980–92.

203.	Vaz S, Thomson A, Cuomo B, Falkmer T, Chamberlain A, Black MH. Co-occurring intellectual disability and autism: Associations with stress, coping, time use, and quality of life in caregivers. Research in Autism Spectrum Disorders. 2021;84.

204.	de Vries M, Verdam MG, Prins PJ, Schmand BA, Geurts HM. Exploring possible predictors and moderators of an executive function training for children with an autism spectrum disorder. Autism. 2018 May;22(4):440–9.

205.	de Vries M, Geurts H. Influence of Autism Traits and Executive Functioning on Quality of Life in Children with an Autism Spectrum Disorder. J Autism Dev Disord. 2015;45(9):2734–43.

206.	Waheed A, Khan MJ, Khurshid M. Effect of behavioral problems of children with autism in their mother’s quality of life. Pakistan Armed Forces Medical Journal. 2020 Dec 15;70(6):1666–70.

207.	Wang H, Hu X, Han ZR. Parental stress, involvement, and family quality of life in mothers and fathers of children with autism spectrum disorder in mainland China: A dyadic analysis. Res Dev Disabil. 2020 Dec;107:103791.

208.	Wang H, Wang Q, Hu X, Han ZR. Mindfulness and Stress Among Parents of Children with Autism Spectrum Disorder in China. J Autism Dev Disord. 2022 May;52(5):2035–45.

209.	Wang Y, Xiao L, Chen RS, Chen C, Xun GL, Lu XZ, et al. Social impairment of children with autism spectrum disorder affects parental quality of life in different ways. Psychiatry Res. 2018 Aug;266:168–74.

210.	Werner S, Shulman C. Subjective well-being among family caregivers of individuals with developmental disabilities: the role of affiliate stigma and psychosocial moderating variables. Res Dev Disabil. 2013 Nov;34(11):4103–14.

211.	Willet M, Dorstyn D, Due C, Li W. Applying Andersen’s Model to Explain Service Use and Quality of Life Among Australian Caregivers of Children with Autism Spectrum Disorder. J Dev Phys Disabil. 2018 June 1;30(3):339–54.

212.	Williams K, Jacoby P, Whitehouse A, Kim R, Epstein A, Murphy N, et al. Functioning, participation, and quality of life in children with intellectual disability: an observational study. Dev Med Child Neurol. 2021 Jan;63(1):89–96.

213.	Ye Y, Deng T, Chen M, Huang B, Ji Y, Feng Y, et al. Relationship between affiliate stigma and family quality of life among parents of children with autism spectrum disorders: The mediating role of parenting self-efficacy. Arch Psychiatr Nurs. 2024 Apr;49:23–31.

214.	Zeng S, Hu X, Zhao H, Stone-MacDonald AK. Examining the relationships of parental stress, family support and family quality of life: A structural equation modeling approach. Res Dev Disabil. 2020 Jan;96:103523.

215.	Zeng S, Zhao H, Hu X, Lee JD, Stone-MacDonald AK, Price ZW. Are we on the same page: A dyadic analysis of parental stress, support, and Family Quality of Life on raising children with autism spectrum disorders. Journal of Developmental and Physical Disabilities. 2021;33(4):599–618.

216.	Zhao M, You Y, Chen S, Li L, Du X, Wang Y. Effects of a Web-Based Parent-Child Physical Activity Program on Mental Health in Parents of Children with ASD. Int J Environ Res Public Health. 2021 Dec 7;18(24):12913.
